# More than presence-absence; modelling (e)DNA concentration across time and space from qPCR survey data

**DOI:** 10.1007/s42519-025-00477-9

**Published:** 2025-08-05

**Authors:** Milly Jones, Eleni Matechou, Diana Cole, Alex Diana, Jim Griffin, Sara Peixoto, Lori Lawson Handley, Andrew Buxton

**Affiliations:** 1 School of Mathematics, Statistics, and Actuarial Science, University of Kent, Cornwallis South, Canterbury, CT2 7NF Kent England; 2 School of Mathematics, Statistics, and Actuarial Science, University of Essex, Wivenhoe Park, Colchester, CO4 3SQ Essex England; 3 Department of Statistical Science, University College London, Gower Street, London, WC1E 6BT England; 4 School of Natural Sciences, University of Hull, Hull, HU6 7RX England; 5 Lake Ecosystems Group, UK Centre for Ecology and Hydrology, Lancaster Environment Centre, Bailrigg, Lancaster, LA1 4AP England; 6 NatureSpace, 22 St Peter’s Street, Stamford Lincolnshire, PE9 2PF England; 7 School of Mathematical Sciences, Queen Mary University of London, Mile End Road, London, E1 4NS England

**Keywords:** Environmental DNA, Quantitative PCR, Bayesian Modelling

## Abstract

Environmental DNA (eDNA) surveys offer a revolutionary approach to species monitoring by detecting DNA traces left by organisms in environmental samples, such as water and soil. These surveys provide a cost-effective, non-invasive, and highly sensitive alternative to traditional methods that rely on direct observation of species, especially for protected or invasive species. Quantitative PCR (qPCR) is a technique used to amplify and quantify a targeted DNA molecule, making it a popular tool for monitoring focal species. Modelling of qPCR data has so far focused on inferring species presence/absence at surveyed sites. However, qPCR output is also informative regarding DNA concentration of the species in the sample, and hence, with the appropriate modelling approach, in the environment. In this paper, we introduce a modelling framework that infers DNA concentration at surveyed sites across time and space, and as a function of covariates, from qPCR output. Our approach accounts for contamination and inhibition in lab analyses, addressing biases particularly notable at low DNA concentrations, and for the inherent stochasticity in the corresponding data. Additionally, we incorporate heteroscedasticity in qPCR output, recognizing the increased variance of qPCR data at lower DNA concentrations. We validate our model through a simulation study, comparing its performance against models that ignore contamination/inhibition and variance heterogeneity. Further, we apply the model to three case studies involving aquatic and semi-aquatic species surveys in the UK. Our findings demonstrate improved accuracy and robustness in estimating DNA concentrations, offering a refined tool for ecological monitoring and conservation efforts.

**Supplementary Information:**

The online version contains supplementary material available at 10.1007/s42519-025-00477-9.

## Introduction

Environmental DNA (eDNA) is DNA that individuals of a species leave behind in the environment. Therefore, eDNA surveys allow monitoring of species in the wild by targeting detection of their DNA in corresponding physical samples, such as water or soil [45]. eDNA is increasingly becoming a standard application in bio-monitoring, both alongside and independently of traditional survey methodologies [35]. This is particularly the case for protected [4] or invasive [44] species, as eDNA surveys can be more cost effective [13, 37] and provide high probabilities of species detection [26, 31] in an inexpensive, and non-invasive survey approach. Therefore, eDNA surveys are quickly becoming a widely employed sampling method for wildlife populations [35], and models for the corresponding data are increasingly being developed [7, 12, 17, 39].

eDNA surveys comprise of three stages: DNA availability in the environment, DNA collection in environmental samples, and DNA analysis of the samples in the laboratory [17]. The amount of DNA available for collection from the environment is expected to vary spatially and/or temporally, and as a function of landscape and site covariates, with additional stochasticity at the individual site level [19]. During DNA collection, across the surveyed site(s), and at one or more time points, a number of samples are collected from the environment. DNA concentrations in collected samples are noisy observations of the DNA concentration in the environment and can be functions of environmental covariates (such as temperature, rainfall, or pH [19]) or technical covariates (such as collection method [5]). DNA analysis typically relies on PCR (Polymerase Chain Reaction), during which the physical samples are divided into technical replicates, and the DNA in each replicate is amplified using appropriate primers. In the quantitative PCR (qPCR) protocol, DNA copies in a sample are successively amplified through several fluorescence-based PCR cycles. The qPCR process results in an exponential amplification curve that measures the fluorescence signal against the PCR cycle number. The threshold cycle (CT) value is then the fractional cycle number at which point the fluorescence of a sample crosses a threshold, which is set by the corresponding software as the point where the fluorescence signal exceeds the background noise but is still within the exponential growth phase. Should a sample’s fluorescence signal surpass the threshold, then the PCR run is said to be successful (positive), and the sample has amplified. In general, samples with higher amounts of DNA concentration are expected to amplify faster (i.e. in an earlier PCR cycle), and hence have lower CT values [41].

qPCR is a widely used method for monitoring targeted species as it can be tailored to the species of interest by designing species-specific primers [35]. When modelling qPCR data, focus is often on inferring and reporting DNA presence/absence at surveyed sites [2, 17] by only using the information on whether each PCR replicate was positive or not. However, the link between CT and the initial DNA concentration in the sample can be ascertained using standards (samples of known concentration run alongside samples collected from the environment). Modelling log-concentration in the standard as the covariate and the CT value as the response gives a straight regression line with negative slope [30]. Comparing the CT values between standards, which have a known DNA concentration, and CT values from collected physical samples allows inferring DNA concentrations for the latter. Indeed, more recently, there has been a greater effort to infer DNA concentration rather than just presence/absence from qPCR data [39]. Internally the CT values are transformed to give estimates of DNA concentration in samples based on the regression line generated by the standards. These values are often then used to fit models investigating effects of covariates on DNA concentrations in the environment in a two stage design rather than a single model propagating uncertainty through all analysis [6, 29]. One stage models linking CT values to DNA concentrations in the environment include Espe et al. [12] and Shelton et al. [39], though these do not account for all error and noise in the data-generating process discussed below.

Despite best and continuously improving field and lab practices, DNA-based surveys will always lead to noisy and error-prone data. In addition to the variation in DNA concentrations at availability and collection stages, CT values themselves are noisy indicators of the amount of DNA in the sample, as results from PCR runs on the same sample and under the same protocols vary. The regression line between CT values and DNA concentration is expected to vary slightly across PCR assays [41]. Additionally, the dispersion of CT values for a given DNA concentration increases as the concentration of DNA in the sample decreases [14, 27] (in other words, CT values are heteroscedastic). Both the variation in the regression line across plates and the CT heteroscedasticity can be seen in Figure 3 for the standards from one of the case studies presented in this paper, but the pattern is expected in all cases. Furthermore, qPCR analyses are only run for a maximum number of cycles, CT.max. Samples with low concentrations of DNA often therefore fail to amplify despite presence of DNA as their fluorescence signal failed to pass the threshold before CT.max elapsed. In this way, qPCR analyses can experience false negative errors at low concentrations of DNA in samples due to this right censoring of CT values. In addition to the natural variation in CT values described above, PCR analyses may suffer from contamination or inhibition. Contamination may occur in lab settings due to the presence of target species DNA outside of collected samples that enter into replicates on PCR plates. Inhibition occurs when PCR inhibitors interact with the PCR amplification process to reduce the efficiency of the reaction, and in extreme circumstances can prevent amplification even if the target sequence of DNA is present [21]. Failure to account for contamination may lead to biased inferences, such as false positive errors (incorrectly inferring that presence of DNA in the sample comes from the environment) or high DNA concentrations in the environment. Similarly, failure to account for inhibition could lead to biased inferences about low DNA concentrations [18] or a false negative error (incorrectly inferring absence of DNA in the sample).

Currently, different ad-hoc measures are taken to deal with suspected contamination or inhibition in samples, PCRs, or plates, but these differ between labs or research protocols, are arbitrary, and result in data loss. Currently, a CT value shift of over 2 cycles [42], 3 cycles [20], or 5 cycles [40], in the IPC (internal positive controls) of the environmental sample can be considered evidence of inhibition. When potential inhibition is identified, the affected sample is often diluted and re-analysed with a correction factor to account for the dilution, however the dilution may also result in a failure of samples to amplify [18]. To account for contamination, often, if there is a detection of DNA in negative controls (field blanks, extraction, or no template controls), then any positive detections associated with the sampling occasion or plate are discarded [18, 22, 37]. This may present a loss of information and wasted effort. Alternatively, rather than discard samples, the maximum average concentration of DNA associated with negative field controls can be used as a baseline amount of DNA to subtract from samples collected in the environment [28]. It has been suggested that increasing the number of samples taken or technical replicates analysed may help make inferences more robust, as in occupancy studies [7, 18], but there is a need for a single framework to estimate DNA concentration across surveyed sites, accounting for contamination and inhibition, without discarding samples or resorting to ad-hoc rules of thumb.

Previous work on modelling false negative errors due to low DNA concentrations includes using right censoring at CT.max [12], or using a hurdle model (modelling the probability of amplification and then distribution of CT values conditional on amplification [39]). Within an occupancy framework, Guillera-Arroita et al. [15] and Griffin et al. [17] account for both false positive and false negative errors by treating the data as binary successes and failures. However, little attention has been paid to accounting for contamination and inhibition while estimating DNA concentrations without discarding samples. Further, whilst the relationship between CT values and log-DNA has been established [30] and modelled [12, 39], few models account for the heteroscedasticity in CT values across log-DNA (Matz et al. [27] do so for qRT-PCR with a Poisson log-normal model). In particular, if accounting for contamination or inhibition in samples, it becomes necessary to understand whether differences in CT values are due to the increased variation at lower concentrations or due to error.

We present a model that links CT to DNA concentration (building from one stage models as in Espe et al. [12]; Shelton et al. [39]), and include a collection stage to model variation in collected DNA in samples across a site, allowing for covariates at both the site and sample collection stages [39]. Additionally, we include a temporal model on the available DNA across sites. Similar models at this stage include work by Shelton et al.[39] who consider a spatially smooth function on log-DNA over their coastal site. Finally, we account for contamination or inhibition of technical replicates at the PCR analysis stage, and allow for these to be identified and incorporated into the model in such a way as to mitigate potential biases in inferred DNA concentration (particularly when DNA concentration is low and so these effects are more keenly felt). We also account and correct for the heteroscedasticity in the distribution of CT values across DNA concentrations.

The paper is structured as follows. The model is presented in Section 2. A simulation study is used in Section 3 to compare, over a range of survey designs, the full model to one model ignoring contamination/inhibition and another ignoring CT variance heterogeneity. We illustrate how the model can be utilised via three case studies in Section 4. The first surveys zebra mussels *(Dreissena polymorpha)* in aquatic systems across the UK. This survey covers twenty sites (five sites across four different aquatic environments - lakes, rivers, canals, and reservoirs), with each site being visited only once between July and August 2021. The second surveys zebra mussels in the River Hull and Eccup Reservoir, with repeat visits to each site once a month from December 2020 to November 2021. The final study surveys great crested newts *(Triturus cristatus)* in eight ponds at the University of Kent campus, with repeat fortnightly visits from February to October 2015.

## Model

The data consist of DNA sampled from the environment at *n* sites and across *T* time points. Let $M_{it}$ be the number of samples taken from the environment at site *i*, $i = 1, \cdots , n$, and time *t*, $t = 1, \cdots , T$. We collect site-specific covariates $X^b$, and sample-specific covariates $X^w$. In the lab, the *m*-th sample from site *i* and time *t* is divided into $K_{imt}$ PCR replicates (also called technical replicates). Each replicates *k* is then analysed on some PCR plate *p*, $p = 1, \cdots , P$, during a PCR run. We denote by $C_{imtk}$ the cycles to threshold (CT) value for replicate *k*. PCR runs have a maximum CT, which we denote CT.max, after which the run is ended. In what follows, *i* indexes the site, *t* the time, *m* the sample, *k* the replicate, and *p* the plate.

**Fig. 1:**
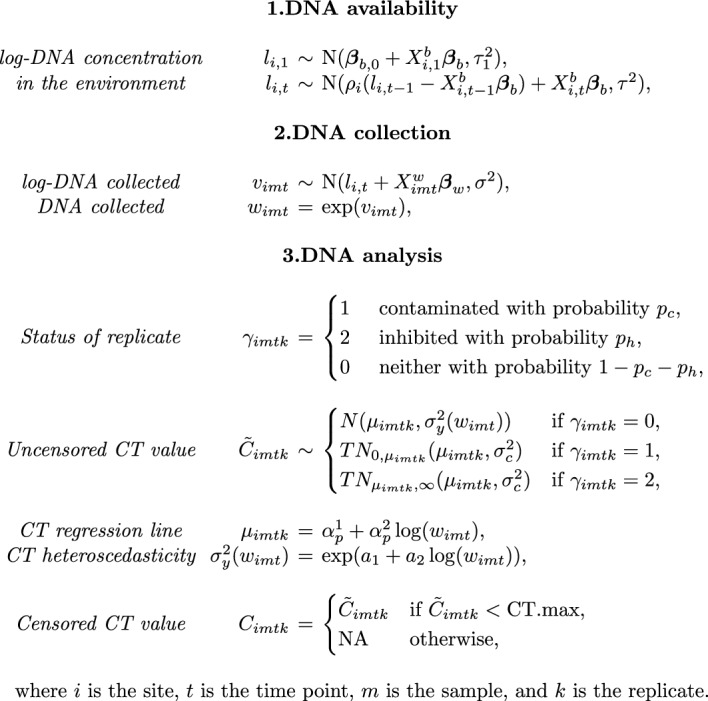
The full model highlighting the three modelling stages: DNA availability, DNA collection, and DNA analysis

Our model (see Figure 1) is divided into 3 stages: DNA availability, DNA collection, and DNA analysis, as is standard for models for DNA-based data of this type [39]. The first stage models the log-DNA concentration at each site and time point, $l_{i,t},$ as a function of site-specific covariates and the DNA concentration at the previous time point. The second stage models the log-DNA concentration in each sample, $v_{imt}$, as a function of the amount of DNA available and of sample-specific covariates. The last stage models $C_{imtk}$ for each replicate. The expected CT values, $\mu _{imtk}$, are a function of the DNA concentration in the sample, where replicates with greater concentrations of DNA are expected to amplify faster. The variability of CT about $\mu _{imtk}$ is also dependent on the DNA concentration in the sample, so that the distribution of CT is heteroscedastic. The variation in CT values decreases as the concentration of CT increases (see for example Figure 3b). A replicate may also experience contamination or inhibition. In the case of contamination, the CT value is expected to be smaller than $\mu _{imtk}$ due to the presence of additional DNA. For inhibition, the CT value is expected to be larger than $\mu _{imtk}$ due to factors interfering with the amplification of DNA. Some replicates may fail to amplify before CT.max elapses, either due to low concentration of DNA or inhibition, and these result in values we denote by NA. The corresponding model for each stage is described below.

**DNA availability** We model $l_{i,t}$, as a latent AR(1) process plus exogenous predictors [34]:

$$\begin{aligned} l_{i,t} \sim \text {N} (\rho _i (l_{i,t-1} - X^b_{i, t-1} {\varvec{\beta }}_b) + X^b_{i,t}{\varvec{\beta }}_b, \tau ^2), \end{aligned}$$

where ${\varvec{\beta }}_b$ is the vector of coefficients for the covariates, $X^b_{i,t}$, of the corresponding sampling occasion, $\tau ^2$ models the noise across time (assumed to be constant across sites), and $\rho _{i}$ is the AR(1) rate coefficient for site *i*. Therefore, we model $l_{i,t}$ as the sum of a latent AR(1) process and the effect of predictors at the time of sampling. The latent AR(1) process is independent of the predictors; in other words $\rho _i$ is the growth term of the latent process, which is obtained by subtracting the predictors from $l_{i,t}$ at a sampling occasion. In order to estimate the temporal terms $\rho _i$, we borrow information across sites using a hierarchical model so that:

$$\begin{aligned} \rho _i&\sim \text {N}(\rho _0, \sigma ^2_\rho ), \hspace{2cm} \text {for } i = 1, \cdots , n, \end{aligned}$$

for $\rho _0$ and $\sigma ^2_\rho$ the mean and noise across sites, respectively.

For $t=1$ (or when $T=1$ so that we only have a single time point), we let:

$$\begin{aligned} l_{i,1} \sim \text {N}({\varvec{\beta }}_{b,0} + X^b_{i,1} {\varvec{\beta }}_b, \tau _1^2), \end{aligned}$$

where $\tau ^2_1$ models the variation across sites at $t=1$ (rather than across time) and ${\varvec{\beta }}_{b,0}$ is the mean log-DNA across sites at $t=1$.

**DNA collection** Given $l_{i,t}$, we then model $v_{imt}$ as follows:

$$\begin{aligned} v_{imt} \sim \text {N}(l_{i,t} + X^w_{imt}{\varvec{\beta }}_w, \sigma ^2), \end{aligned}$$

where ${\varvec{\beta }}_w$ is the vector of covariate coefficients for the sample-specific covariates $X^w_{imt}$, and $\sigma ^2$ is the noise across samples. Unlike Diana et al. [10], we do not explicitly model false negative (inhibition) or positive (contamination) errors at the sample collection stage, and assume that the noise term $\sigma ^2$ adequately accounts for these events. We return to this assumption in Section 5.

**DNA analysis** We assume that DNA in the sample is uniformly distributed, so that each replicate $k = 1, \cdots , K_{imt}$ from the same sample has concentration $w_{imt} = \exp (v_{imt})$. To account for the replicates that fail to amplify before CT.max, we use a right censoring model for the CT values [12]. We first present a model for the uncensored CT values, $\tilde{C}_{imtk}$, and then censor these at CT.max to get the observed CT values $C_{imtk}$.

We model $\tilde{C}_{imtk}$ as linear in log-DNA, with plate-specific regression coefficients, with log-variance as linear in log-DNA, and allow for contamination and inhibition with a mixture model. For the mixture model, we introduce a latent indicator variable, $\gamma _{imtk}$, such that:

$$\begin{aligned} \gamma _{imtk} = {\left\{ \begin{array}{ll} 1 \hspace{1cm} \text {replicate contaminated with probability } p_c,\\ 2 \hspace{1cm} \text {replicate inhibited with probability } p_h,\\ 0 \hspace{1cm} \text {neither with probability } 1- p_c-p_h, \end{array}\right. } \end{aligned}$$

where $p_c$ is the probability a replicate is contaminated and $p_h$ is the probability a replicate is inhibited. We assume that a replicate cannot be simultaneously contaminated and inhibited, and that $p_c$ and $p_h$ are equal across all PCR runs.

Conditional on $\gamma _{imtk}$, $\tilde{C}_{imtk}$ is then modelled as:

$$\begin{aligned} \tilde{C}_{imtk}&\sim {\left\{ \begin{array}{ll} \textrm{N}(\mu _{imtk}, \sigma ^2_y(w_{imt})) &  \text {if } \gamma _{imtk} = 0,\\ \textrm{TN}_{0, \mu _{imtk}}(\mu _{imtk}, \sigma ^2_c) &  \text {if } \gamma _{imtk} = 1,\\ \textrm{TN}_{\mu _{imtk}, \infty }(\mu _{imtk}, \sigma ^2_c) &  \text {if } \gamma _{imtk} = 2,\\ \end{array}\right. }\\ \mu _{imtk}&= \alpha ^1_{p} + \alpha ^2_{p} \log (w_{imt}), \end{aligned}$$

where $\hbox {TN}_{a,b}(\mu , \sigma ^2)$ is the normal distribution with mean $\mu$ and variance $\sigma ^2$, truncated between *a* and *b*. The plate-specific regression coefficients, $\alpha ^1_p$ and $\alpha ^2_p$, share information across the *P* plates using a hierarchical model:

$$\begin{aligned} \alpha ^1_p&\sim \text {N}(\alpha ^1_0, \sigma ^2_{\alpha }) \hspace{1cm} \text {for } p = 1, \cdots , P, \\ \alpha ^2_p&\sim \text {N}(\alpha ^2_0, \sigma ^2_{\alpha }) \hspace{1cm} \text {for } p = 1, \cdots , P. \end{aligned}$$

When $\gamma _{imtk} = 0$, to account for the heteroscedasticity of CT values, we follow the method of Cook and Weisberg [9] and write:

$$\begin{aligned} \log (\sigma _y^2(w_{imt})) = a_1 + a_2 \log (w_{imt}), \end{aligned}$$

where $a_1$ and $a_2$ are the regression coefficients for log-variance against log-DNA.

The truncated normal distribution is used to capture the expected behaviour of contaminated ($\gamma _{imtk} = 1$) and inhibited ($\gamma _{imtk}=2$) replicates. For example, as discussed earlier, we expect contaminated replicates to amplify faster than the expected value $\mu _{imtk}$ (the expected value when no contamination is present). In both cases, we take some large $\sigma _c^2$ (in practice we take $\sigma _c$ about the order of CT.max) which enables $\tilde{C}_{imtk}$ to take a wide range of values within the appropriate interval. We expect contamination or inhibition to be rare, or for the distributions of affected samples to vary widely across sampling occasions and PCR runs. This approach (similar to a variance-inflation method [25]) means that we need not learn the distribution of contaminated or inhibited replicates as in a mean-shift method. The mixture model then allows for $p_c$ and $p_h$ to be estimated separately.

Finally, given $\tilde{C}_{imtk}$ and CT.max, the observed $C_{imtk}$ is modelled as:

$$\begin{aligned} C_{imtk} = {\left\{ \begin{array}{ll} \tilde{C}_{imtk} \hspace{1cm} &  \text {if } \tilde{C}_{imtk} < \text {CT.max},\\ \text {NA} \hspace{1cm} &  \text {otherwise.} \end{array}\right. } \end{aligned}$$

On each plate *p*, alongside replicates from samples collected in the environment, are standards (replicates of known amount of log-DNA). Analogous to the samples collected during sampling occasions, we denote by $w^{\star }_{s}$ the amount of DNA, $C^{\star }_{s}$ the CT, and $p^{\star }_s$ the plate in which standard *s* is analysed. The DNA analysis model for standards is the same as for the environmental replicates, except $w^{\star }_s$ is known and does not need to be learnt. In this way, the standards help inform the model PCR analysis parameters (CT regression, CT heteroscedasticity, and probability of contamination and inhibition parameters).

**Fig. 2:**
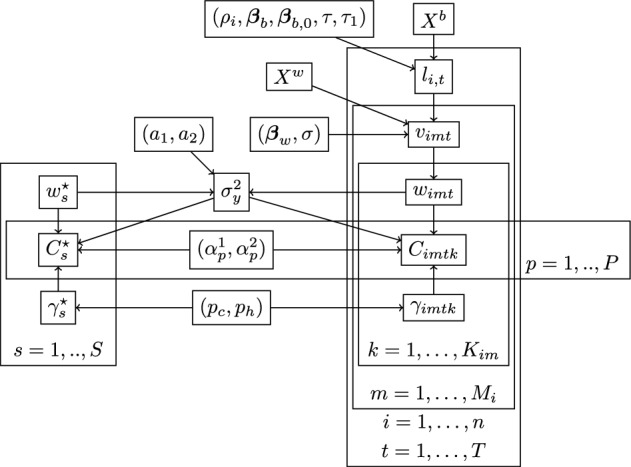
Directed acyclic graph representing the relationships between the variables in the model

We summarise the full model in Figure 1, highlighting the different analysis stages for samples collected from the environment. In Figure 2, we present the directed acyclic graph (DAG) of the full model (including the analysis of standards), showing the relationships between variables.

We implemented the model in NIMBLE [11] in R [32], and all results presented in the paper are obtained using NIMBLE. Model code is available on https://github.com/millyljones/Spatio-temporal-eDNA/tree/main. We include a constraint within the MCMC that $p_c + p_h < 1 - (p_c + p_h)$. In other words, the probability a replicate is contaminated or inhibited cannot be greater than the probability that it is not so. At each iteration of the MCMC, if proposed probabilities $p_c$ and $p_h$ violate this constraint, then both the proposed probabilities are rejected.

## Simulation Study

Ignoring false positive and false negative errors can have an impact on inference of model parameters. For example, Buxton et al. [7] found that ignoring false positive errors in an occupancy study resulted in overestimation of occupancy probabilities. They also found that increasing replication (both in terms of number of samples *M* and technical replicates *K*) reduced bias and posterior credible interval (PCI) width for model parameters in their occupancy study. In this section, we present a simulation study that investigates the effect of ignoring contamination and inhibition at the PCR analysis stage for DNA concentration studies. We consider a range of study designs (varying the number of samples *M* and replicates *K*) to compare their effect on the estimation of model parameters. We also investigate the effect of ignoring CT heteroscedasticity, as this has not been considered by previous models.

Let Model 1 be the full model described in Figure 1. Let Model 2 be as Model 1, except that instead of modelling variance of CT values using $\sigma _y^2(w_{imt})$, the variance of CT values, $\sigma ^2_P$, is held constant across the plate the replicates are analysed on. Let Model 3 be as Model 1, except that we take $p_c = p_h = 0$, so that we ignore contamination and inhibition.

Across the simulations, we assume that there are $n=10$ sites, each visited $T = 20$ times. All samples from a single sampling occasion are analysed on a single, distinct plate. For the standards, on each plate, we take $K^{\star } = 3$ replicates of seven concentrations, $3\times 10^z$, $z = 1, \cdots , 7$. We consider a range of sample designs. We consider taking $M = 1, 2, 5,$ or 10 samples at each site, and then consider using $K = 1, 2, 5, \text {or} 10$ replicates per sample in the analysis. At each site we observe 2 covariates, one continuous ($\sim$N(0,1)) and one binary ($\sim$Bern(0.5)), and set ${\varvec{\beta }}_b = (1, -1)$ respectively. With each sample we also observe 2 covariates, one continuous ($\sim$N(0,1)) and one binary ($\sim$Bern(0.5)), and set ${\varvec{\beta }}_w = (1, -1)$ respectively. For each contaminated replicate, we model the amount of contamination, $\lambda$, using a normal distribution with mean $3\times 10^3$ and standard deviation 100. $\lambda$ is then added to the amount of DNA in the replicate, $w_{imt}$. For inhibited replicates, we delay the amplification process proportionately to the amount of DNA in the sample. We let the expected CT value for inhibited samples indicate that the DNA concentration is 90% lower than the true amount. We discuss the choice of distributions for contamination and inhibition in Section 5. For the first time point at each site, we let ${\varvec{\beta }}_{b,0} = 6$, and then let $\rho _i = 1$ for all sites. The variances $\tau ^2, \tau ^2_1, \sigma ^2 = 1$, and the CT variance parameters are set to $a_1 = 0.2, a_2 = -0.25$. The plate regression parameters $\alpha ^1_p$ and $\alpha ^2_p$ are drawn from $\text {N}(44, 0.1)$ and $\text {N}(-1.7, 0.01)$ respectively. The maximum cycle number CT.max is set to 40.

In the first set of simulations, we let $(p_c, p_h) = (0.05, 0.1)$, and in the second set we let $(p_c, p_h) = (0.01, 0.02)$. These two cases compare the model’s performance under substantial and then very small contamination and inhibition. We take the probability of inhibition to be greater than contamination as this is what is more commonly observed in practice. For example Griffin et al. [17] found false negative and false positive error probabilities of 19% and 5% during the laboratory analysis stage. We also find that $p_h$ is generally greater than or similar to (with overlapping PCIs) $p_c$ in our case studies presented in Section 4.

For each simulation we generate and analyse $N = 100$ data sets. In Section 3.1, we compare the posterior summaries of $l_{i,t}$ and other model parameters across the $N = 100$ data sets. Details about prior distributions and MCMC parameters for each simulation can be found in Section S1.

### Simulation results

We denote by $l_{i,t, j}$ and $\tilde{l}_{i,t, j}$ the true and posterior mean log-DNA concentrations respectively for site *i*, at time *t*, in simulation *j*. For *N* simulations, we compute the mean square error (MSE) as:

$$\begin{aligned} \text {MSE} = \frac{1}{NnT} \sum _{j=1}^N \sum _{i=1}^n \sum _{t=1}^T (l_{i,t,j} - \tilde{l}_{i,t,j})^2. \end{aligned}$$

We denote by $\theta _j$ and $\tilde{\theta _j}$ the true value and the posterior mean of some parameter in the model for simulation *j*. Then the mean bias (MB) is:

$$\begin{aligned} \text {MB} = \frac{1}{N} \sum _{j=1}^N (\tilde{\theta _j} - \theta _j) \end{aligned}$$

The 95% PCIs are computed given the 2.5% and 97.5% quantiles of the posterior distribution.

**Table 1:** Mean square error (MSE), mean range of 95% PCIs (R), and mean coverage of $l_{i,t}$ (C) across Model 1 (full model), Model 2 (constant CT variance), and Model 3 (ignoring contamination and inhibition) under different sampling designs. Probability of contamination and inhibition $(p_c, p_h) = (0.05, 0.1)$ and $(p_c, p_h) = (0.01, 0.02)$

$$(p_c, p_h) = (0.05, 0.1)$$									
	Model 1			Model 2			Model 3		
M = 1	MSE	R	C	MSE	R	C	MSE	R	C
K = 1	1.212	3.726	0.930	1.219	3.636	0.922	1.493	3.976	0.928
K = 2	0.858	3.312	0.940	0.842	3.183	0.931	1.071	3.510	0.933
K = 5	0.776	3.031	0.933	0.790	2.892	0.923	1.012	3.182	0.924
K = 10	0.746	3.013	0.942	0.783	2.836	0.920	1.577	3.261	0.938
M = 2	MSE	R	C	MSE	R	C	MSE	R	C
K = 1	0.715	2.953	0.936	0.711	2.875	0.932	0.898	3.15	0.934
K = 2	0.605	2.636	0.939	0.612	2.535	0.931	0.900	2.797	0.925
K = 5	0.442	2.427	0.948	0.502	2.320	0.930	0.587	2.579	0.936
K = 10	0.498	2.402	0.943	0.589	2.263	0.919	4.855	2.898	0.931
M = 5	MSE	R	C	MSE	R	C	MSE	R	C
K = 1	0.424	2.216	0.936	0.432	2.155	0.929	0.673	2.427	0.923
K = 2	0.312	1.960	0.946	0.330	1.865	0.933	0.492	2.146	0.935
K = 5	0.249	1.790	0.950	0.304	1.709	0.932	1.938	2.179	0.940
K = 10	0.261	1.750	0.946	0.361	1.651	0.921	12.439	2.577	0.895
M = 10	MSE	R	C	MSE	R	C	MSE	R	C
K = 1	0.268	1.761	0.937	0.284	1.696	0.930	0.601	1.975	0.921
K = 2	0.205	1.506	0.945	0.243	1.430	0.930	0.444	1.781	0.946
K = 5	0.174	1.408	0.946	0.265	1.338	0.919	10.236	2.348	0.896
K = 10	0.193	1.380	0.944	0.330	1.301	0.910	18.475	2.385	0.817

$$(p_c, p_h) = (0.01, 0.02)$$									
	Model 1			Model 2			Model 3		
M = 1	MSE	R	C	MSE	R	C	MSE	R	C
K = 1	0.914	3.309	0.934	0.915	3.253	0.931	0.975	3.391	0.937
K = 2	0.732	3.156	0.945	0.709	3.057	0.941	0.798	3.203	0.944
K = 5	0.682	2.962	0.935	0.722	2.840	0.922	0.773	3.001	0.932
K = 10	0.648	2.925	0.941	0.706	2.774	0.922	0.730	2.958	0.936
M = 2	MSE	R	C	MSE	R	C	MSE	R	C
K = 1	0.560	2.599	0.944	0.560	2.555	0.940	0.611	2.675	0.944
K = 2	0.495	2.497	0.945	0.514	2.414	0.937	0.568	2.562	0.942
K = 5	0.510	2.421	0.944	0.543	2.302	0.929	0.587	2.467	0.939
K = 10	0.486	2.367	0.940	0.545	2.238	0.917	0.966	2.471	0.937
M = 5	MSE	R	C	MSE	R	C	MSE	R	C
K = 1	0.323	1.940	0.943	0.341	1.886	0.936	0.431	2.016	0.937
K = 2	0.315	1.869	0.944	0.329	1.788	0.932	0.441	1.927	0.939
K = 5	0.268	1.756	0.948	0.350	1.673	0.925	0.747	1.859	0.942
K = 10	0.254	1.741	0.943	0.371	1.638	0.909	2.382	2.099	0.937
M = 10	MSE	R	C	MSE	R	C	MSE	R	C
K = 1	0.208	1.534	0.948	0.228	1.480	0.938	0.341	1.614	0.936
K = 2	0.205	1.436	0.948	0.231	1.372	0.930	0.331	1.517	0.941
K = 5	0.178	1.382	0.945	0.243	1.312	0.919	1.224	1.599	0.938
K = 10	0.185	1.350	0.944	0.320	1.276	0.907	7.419	2.055	0.909

Table 1 shows results for MSE, mean width of 95% PCIs, and corresponding mean coverage for $l_{i,t}$ in the cases $(p_c, p_h) = (0.05, 0.1)$ and $(p_c, p_h) = (0.01, 0.02)$. Tables S2 and S3 show results for MB, mean width of 95% PCIs, and mean proportion of PCIs containing zero for the site and sampling coefficients ${\varvec{\beta }}_b$ and ${\varvec{\beta }}_w$ for $(p_c, p_h) = (0.05, 0.1)$ and $(p_c, p_h) = (0.01, 0.02)$ respectively. Tables S4 and S5 show the same for the log-variance parameters $a_1, a_2$, and the probabilities $p_c$ and $p_h$.

In Table 1 we can see that for Model 1 the larger improvements in MSE and PCIs come when increasing either M or K from 1 to 2. In other words, replication in either collection of samples or in the PCR analysis yields improvements in both the posterior means and credible intervals of $l_{i,t}$. Increasing M or K beyond 2 decreases MSE and narrows PCIs, but with diminishing returns. Increasing the number of samples M has a more considerable effect on the MSE than increasing K, but comes with an increased cost of effort in the field. Models 2 and 3 have lower coverage on average than Model 1. Model 2 underestimates the variability in the CT values, and so does not account for the full uncertainty in the data-generating process, leading to narrower PCIs that have smaller coverage. In Table 1, where $(p_c, p_h) = (0.05, 0.1)$, Model 3 does not account for errors in the PCR stage of analysis, and so fails to correctly account for contamination and inhibition, and so has much higher MSE, wider PCIs, and lower coverage. In fact for Model 3 the MSE increases for increasing K as there is more chance for samples to have a replicate experience either contamination or inhibition, leading to more false positive and negative errors, and greater bias in the analysis. Under the simulation parameters we have investigated, the effect of ignoring contamination and inhibition leads to worse outcomes than the effect of ignoring CT heteroscedasticity. We return to this in Section 5 as other simulation parameters may lead to different conclusions.

In Tables S2 and S3, we can see that increasing M and K reduces mean bias in ${\varvec{\beta }}_b$ and ${\varvec{\beta }}_w$, and reduces the width of PCIs, increasing power to detect important covariate effects. As with log-DNA, the improvement when increasing M is greater than when increasing K, but at the cost of greater effort in the field. Under our simulation parameters, where binary coefficients (${\varvec{\beta }}_b[2], {\varvec{\beta }}_w[2]$) are drawn from a Bernoulli distribution with probability 0.5, then a greater amount of replication is needed to detect covariate effects than for the continuous covariates. Model 1 generally has the smallest bias in the mean values of the covariate coefficients, and Model 3 generally has the largest bias and widest credible intervals (though when $p_c$ and $p_h$ are small then this difference is smaller).

In Tables S4 and S5, for both the high and low contamination/inhibition cases, and across the survey designs, there is underestimation of the contamination probability $p_c$ in Model 1. The model’s ability to detect contaminated replicates relies on the concentration of added DNA being high enough to considerably increase the CT value of that replicate. The higher the concentration of the DNA in a sample, the smaller the effect of contamination, and so in our simulation not all contamination is detectable. So this underestimation was to be expected. There is similarly often a small negative bias in the probability of inhibition $p_h$, though due to the way these replicates were simulated, the model was able to detect these more often. As a consequence of the small underestimation of probabilities $p_c$ and $p_h$, the intercept on the log-variance, $a_1$, is slightly overestimated, as replicates that were contaminated or inhibited, but not labelled as such, have pushed up the variance slightly. Increasing the number of technical replicates *K* does help to reduce the positive bias in $a_1$, and generally also improves the posterior means of $a_2, p_c,$ and $p_h$ whilst reducing the width of the 95% PCIs. In Table S5, where $p_c$ and $p_h$ are negligible, then Model 1 still provides reasonable posterior means for $p_c$ and $p_h$, so the model still performs well if levels of contamination and inhibition are very small. Model 2 generally has comparable posterior means and PCIs to Model 1 for $p_c$ and $p_h$. Model 3 has large biases in estimates of $a_1$ and $a_2$, even in the case where $p_c$ and $p_h$ are low.

## Case studies

We consider three case studies: zebra mussels (*Dreissena polymorpha*) across multiple sites but a single time point in Section 4.1; zebra mussels across two sites and multiple time points in Section 4.2; great crested newts (*Triturus cristatus*) at a single site and multiple time points in Section 4.3.

### Zebra mussels: single time point

eDNA samples were collected from n = 20 sites within England, during July and August of 2021, with zebra mussels (*Dreissena polymorpha*) as the target species. At each site, M = 10 samples were collected and sampling locations were chosen based on safety and accessibility to the water. For running waters, like canals and rivers, samples were collected over a 1 km stretch and evenly spaced, when possible, while for standing waters, such as lakes and reservoirs, sampling was conducted around the perimeter of the site. A full list of the sampled sites can be found in Table S6.

Water was filtered through an enclosed NatureMetrics filter using a 100 mL luer-lock syringe until the filter clogged, and DNA was preserved with Longmire’s buffer. The samples were extracted using a modified DNeasy Blood & Tissue kit (Qiagen) and tested for inhibition with the TaqMan Exogenous Internal Positive Control (Fisher Scientific). These tests did not indicate evidence of inhibition in the samples. Species-specific qPCRs were conducted using, with minor modifications, the cytochrome b assay described in Gingera et al. [16] on a StepOnePlus Real-Time PCR machine. Samples were considered positive if their signal intersected the threshold line defined by the software, and the cycle at which that intersection occurred corresponded to the CT value of the sample.

At each site and sampling location, water chemistry data such as water temperature, pH, turbidity and conductivity were recorded using a portable meter (HI-98130, Hanna Instruments), and calcium levels were obtained using a calcium meter (LAQUATwin Calcium Ion Ca-11 meter, Camlab). In addition, the depth at each sampling point was also recorded, as well as information on substrate type, which was divided into four categories - boulders (B), gravel (G), silt (S), and sand (SA).

Each sample was analysed in $K = 6$ replicates. All replicates from the same site were analysed in the same PCR plate, therefore $p = i$. For the standards, on each plate, we take $K^{\star } = 3$ replicates of seven concentrations, $3\times 10^z$ copies/$\mu$L, $z = 1, \cdots , 7$.

**Fig. 3:**
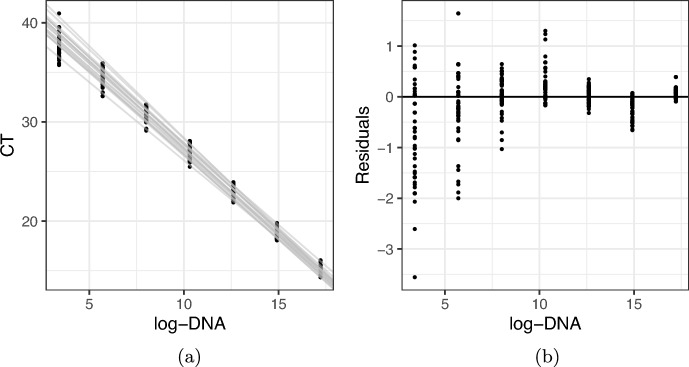
Zebra mussels: single time point. (a) CT values from standards against log-DNA in the standard. Each line is the linear fit of CT against log-DNA for each of the 20 plates (with each plate corresponding to a single site) used to analyse the samples. (b) Residuals of the linear regression with CT as the response and log-DNA concentration as the covariate

Figure 3a demonstrates the linear relationship between log-concentration and CT for the standards by fitting a simple regression for each of the 20 plates (linear modelling using dglm() [36]). The fitted lines show that the PCR efficiency varies slightly between PCR runs, motivating the hierarchical model on the plate-specific regression coefficients. Figure 3b shows that the spread of the residuals of the fitted CT values increases as the log-concentration in the standards decreases, and we account for this heteroscedasticity by modelling CT variance conditionally on log-DNA, as described in Section 2.

The 20 sites were taken from 4 different environments: river, reservoir, lake and canal (with 5 sites for each environment). We use the environment as a covariate for DNA concentration at the site. The covariates included in the model for DNA collection were volume, temperature, calcium, depth, and substrate. Details of model implementation can be found in Section S2.1.2 and details of prior distributions are included in Table S7.

**Fig. 4:**
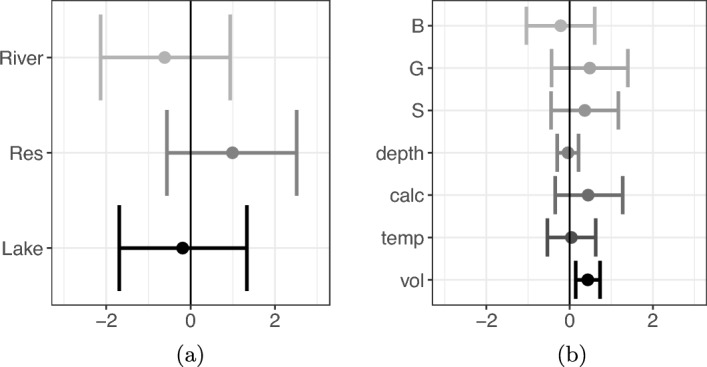
Zebra mussels: single time point. Posterior means (circles) and 95% PCIs (bars) of (a) site-specific covariate coefficients and (b) sample-specific covariate coefficients. (a) Covariates are: environment types (river, reservoir (Res), and lake). (b) Covariates are: substrate types (boulders (B), gravel (G), and silt (S)), depth, calcium (calc), temperature (temp), and volume (vol)

**Fig. 5:**
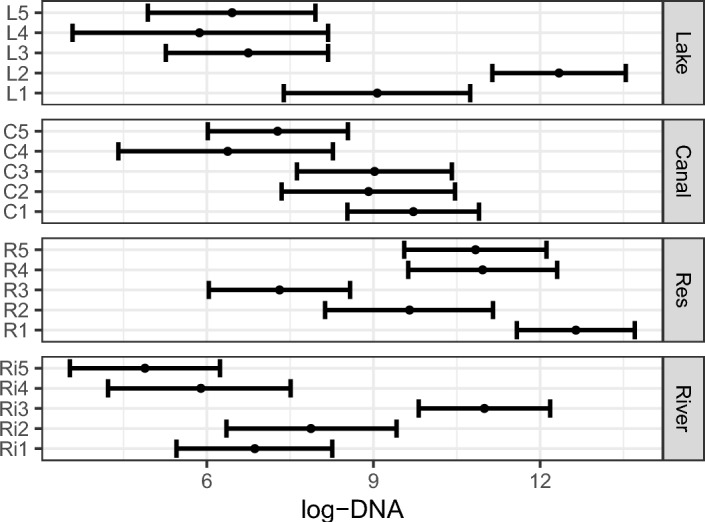
Zebra mussels: single time point. Posterior means (circles) and 95% PCIs (bars) of the log-DNA (copies/$\mu$L) concentration across the 20 sites. The environments for each site are shown on the right label. See Table S6 for the names of sites corresponding to the labels on the left

Results for the posterior distributions of covariate coefficients ${\varvec{\beta }}_b$ and ${\varvec{\beta }}_w$ are reported in Figure 4. Figure 4b shows that, as expected, volume of water filtered has a positive effect on the collection rate of DNA. For the environment types shown in Figure 4a and the other environmental factors shown in Figure 4b, the inclusion of zero within the 95% PCIs of coefficients suggest that the data do not provide strong evidence that these covariates have a non-zero effect. The results for the log-DNA at each site are shown in Figure 5. There is substantial variability in inferred DNA concentrations between sites of the same environmental type (though the canals show the smallest between-site variation). The difference between these DNA concentrations is likely due to site-specific environmental characteristics that were not recorded or used in the model. Canals were the most consistent in terms of habitat (for example in substrate type) amongst the environment types, which may explain the smaller amount of variation between canals. For lakes L1 and L2 (Eight Acre Lake and Farnham Lake respectively), the sampling area was smaller than the other lakes (due to accessibility restrictions), and for L2 in particular, almost all sampling was near pontoons where recreational activities occur, which is a known vector for dispersal for this species. As a result, the area from which the samples were collected likely contained high biomass of species, resulting in higher DNA availability when compared to the other lakes. This was further evidenced by the observation of numerous live and deceased organisms at the sampling locations at Farnham Lake (L2). Similarly, DNA concentrations for Ri3 (River Thames) are much higher than the other rivers in the study likely because the Thames has a higher presence of boat traffic and recreational activities.

Table 2 shows the posterior means and 95% PCIs for the CT variance parameters, $a_1, a_2$, the probabilities of contamination and inhibition, $p_c$, and $p_h$, and the DNA availability and collection standard deviations, $\tau$ and $\sigma$.

**Table 2:** Zebra mussels: single time point. Posterior means and 95% PCIs of variance parameters, $a_1, a_2$, probabilities of contamination and inhibition $p_c, p_h$, and DNA availability and collection standard deviations $\tau$ and $\sigma .$

Parameter	Mean	95% PCI
$a_1$	0.535	(0.308, 0.756)
$a_2$	-0.398	(-0.419, -0.377)
$p_c$	0.019	(0.013, 0.027)
$p_h$	0.011	(0.006, 0.018)
$\tau _1$	2.090	(1.492, 2.940)
$\sigma$	1.399	(1.260, 1.554)

The posterior means for $p_c$ (0.019 [0.013, 0.027]) and $p_h$ (0.011 [0.006, 0.018]) are very small. These probabilities are similar to the settings used in the second set of simulations where $(p_c, p_h) = (0.01, 0.02)$. The results in Table 1 show that ignoring contamination and inhibition (even when these are small), or ignoring CT heteroscedasticity leads to an increase in MSE and lower nominal coverage particularly when *M* and *K* are large, as we have in this case study.

### Zebra mussels: multiple time points

eDNA samples were collected from n = 2 sites in Yorkshire (England), every month, between December 2020 and November 2021, where the target species was the zebra mussel (*Dreissena polymorpha*), as in Section 4.1. At each time point (i.e. month) and each site, M = 10 samples were collected from the shoreline, with sampling locations chosen based on safety and accessibility to the water. At Eccup Reservoir, sampling locations were selected to maximise the perimeter of the reservoir sampled, while at the River Hull samples were collected over a 1 kilometre stretch (i.e. collecting one sample approximately every 100 meters) in a publicly accessible area. Sampling locations were always the same for each time point at both sites.

At each sampling occasion, 2L water samples were collected into a sterile plastic bottle, stored in a sterile cool box with ice packs and transported to the laboratory on the same day of collection. All water samples were vacuum-filtered within 24h of collection in a dedicated laboratory, using two 0.45 $\mu$m cellulose filters (47 mm, Cytiva Whatman Mixed Cellulose Ester Membranes; Fisher Scientific, UK) per sample. The volume filtered for each sample was recorded, and filters were stored at -$20^\circ$C until DNA extraction. Samples were extracted using the water protocol described in Sellers et al. [38], and tested for inhibition with the TaqMan Exogenous Internal Positive Control (Fisher Scientific). This test did not indicate evidence of inhibition in the samples. Species-specific qPCR reactions were performed following the protocol described in Section 4.1.

At each time point and at each of the ten sampling locations at both sites, water chemistry information (temperature, pH, turbidity, conductivity and calcium) was recorded as described in Section 4.1. Calcium data in December, and turbidity and conductivity data in February, are missing due to technical problems with the probes in those months. Water levels were also recorded each month at both sites, by checking a reverse water depth gauge board installed at Eccup Reservoir, and by retrieving data from a monitoring station close to the sampling locations at the River Hull.

Each sample was analysed in $K=6$ technical replicates. All the replicates from the same site and same time point were analysed in the same PCR plate for a total of $P = 24$ plates. For the standards, on each plate, we take $K^{\star } = 3$ replicates of seven concentrations, $3\times 10^z$ copies/$\mu$L, $z = 1, \cdots , 7$. Figures exploring the linear fit between log-DNA in standards and CT value, and the residuals, can be found in Figure S1. The covariates included in the model for DNA collection were volume, pH, and calcium. Details of model implementation can be found in S2.2.2, and details of prior distributions are included in Table S9.

**Fig. 6:**
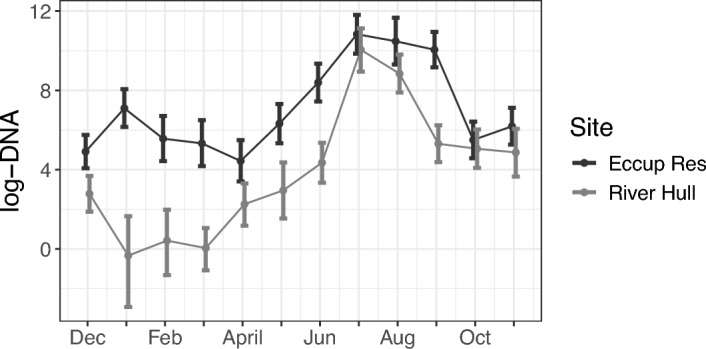
Zebra mussels: multiple time points. Posterior means (circles) and 95% PCIs (bars) of the log-DNA (copies/$\mu$L) concentrations available at Eccup Reservoir and River Hull between December 2020 and November 2021

Results for the posterior distributions of covariate coefficients ${\varvec{\beta }}_w$ are reported in Table 3. We can see that, as in Section 4.1, volume has a positive impact on DNA collection rate. The inclusion of zero within the 95% PCIs of the other covariate coefficients suggests the data do not provide enough evidence these covariates have a non-zero effect on the amount of DNA collected given the amount of DNA available. Figure 6 shows the log-DNA means and 95% PCIs for the two sites. Eccup Reservoir on average has higher log-DNA than the River Hull. In flowing waters, such as rivers, there is a larger dispersion and dilution effect of DNA compared to standing water, such as in reservoirs, which generally leads to lower DNA concentrations. In both sites, the log-DNA experiences a peak in the summer months before dropping over the autumn period. The end of spring and the summer period corresponds to the species’ reproductive season, and as such DNA availability increases.

**Table 3:** Zebra mussels: multiple time points. Mean and 95% PCIs for the sample covariate coefficients (volume, pH, and calcium)

Covariate	Mean	95% PCI
volume	0.401	(-0.034, 0.844)
pH	-0.032	(-0.298, 0.235)
calcium	0.160	(-0.445, 0.787)

Table 4 shows the posterior means and 95% PCIs for the CT variance parameters, $a_1, a_2$, the probabilities of contamination and inhibition, $p_c, p_h$, the DNA availability AR(1) terms $\rho _1, \rho _2$, and the DNA availability and collection standard deviations, $\tau$, $\tau _1,$ and $\sigma$. As with the previous case study (Section 4.1), the posterior means for $p_c$ (0.004 [0.002, 0.008]) and $p_h$ (0.028 [0.018, 0.039]) are very small and are similar to the settings used in the second set of simulations where $(p_c, p_h) = (0.01, 0.02)$. Table 1 shows that ignoring contamination and inhibition (even when these are small), or ignoring CT heteroscedasticity leads to an increase in MSE and lower nominal coverage particularly when *M* and *K* are large, as we have in this case study.

**Table 4:** Zebra mussels: multiple time points. Posterior means and 95% PCIs of variance parameters, $a_1, a_2$, probabilities of contamination and inhibition $p_c, p_h$, DNA availability AR(1) terms $\rho _1, \rho _2$, and DNA availability and collection standard deviations $\tau$, $\tau _1$, and $\sigma .$

Parameter	Mean	95% PCI
$a_1$	1.328	(1.150, 1.505)
$a_2$	-0.406	(-0.424, -0.387)
$p_c$	0.004	(0.002, 0.008)
$p_h$	0.028	(0.018, 0.039)
$\rho _1$	0.973	(0.787, 1.155)
$\rho _2$	0.900	(0.613, 1.177)
$\tau$	2.279	(1.665, 3.134)
$\tau _1$	1.113	(0.536, 2.386)
$\sigma$	1.242	(1.122, 1.381)

### Great crested newts

eDNA samples were collected from a single site comprising of 8 ponds in close proximity to each other at the University of Kent campus every 14 days, between 26 February and 22 October 2015. The target species was great crested newts (*Triturus cristatus*). At each sampling occasion a number of samples were taken (*M* varying between 8 and 24 samples on each occasion), and then analysed in $K=8$ replicates.

Data collection is described in detail in Buxton et al. [6]. Ethanol precipitation eDNA collection method was used alongside 0.7$\mu$m glass-microfiber syringe filters and 0.7$\mu$m cellulose acetate syringe filters. Ethanol precipitation followed methodologies outlined in Biggs et al. [4], collecting 0.09L of sample water, while the two filter methods up to 1L of water was filtered stopping at the point a filter became blocked.

DNA was extracted using Qiagen (R) DNeasy Blood and tissue kits following the protocols outlined in Buxton et al. [6], with qPCR conducted on a Biorad CFX connect Real-Time PCR machine using the primers and hydrolysis probe published in Thomsen et al. [43] and PCR assay and cycle conditions published by Biggs et al. [4]. qPCR standards were created from a serial dilution of a great crested newt tissue extract, quantified using a Qubit®  2.0 flurometer (Life TechnologiesTM, Carlsbad, California, USA) with Double Stranded DNA High Sensitivity Kit following manufacturers’ instructions, qPCR negative controls were also included in each run.

A total of 28 plates were used, where each plate consisted only of samples collected on the same sampling occasion, though samples from that occasion may take up several plates. On each plate, there were $K^\star = 3$ replicates of 3 concentrations, ranging between 0.03 and 7.9 $\mu$g/mL. Figures exploring the linear fit between log-DNA in standards and CT value, and the residuals of the corresponding linear regression, can be found in Figure S2.

The covariates included for DNA availability were mean weekly temperature ($^\circ$C) and mean weekly rainfall (mm). The three DNA collection methods were included as covariates on collection: Ethanol (E) and Glass Microfiber (GF), using Cellulose (C) as the reference level. The pond number of each sample was also included as a covariate on DNA collection (with pond 1 as the reference level). Details of model implementation can be found in S2.3.2, and details of prior distributions are included in Table S11.

**Fig. 7:**
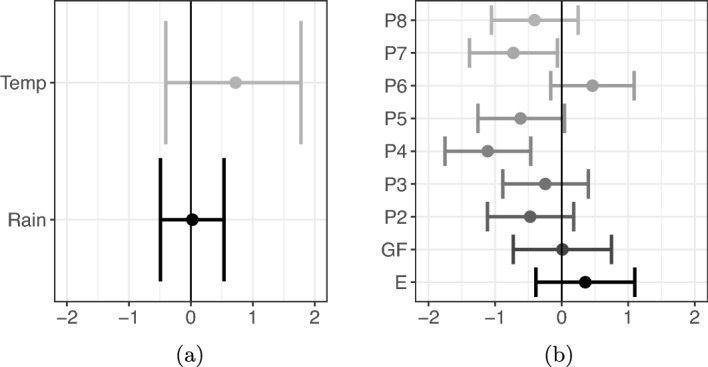
Great crested newts: Posterior means (circles) and 95% PCIs (bars) of (a) site-specific covariate coefficients and (b) sample-specific covariate coefficients. (a) Covariates are mean weekly temperature (Temp), mean weekly rainfall (mm) (Rain). (b) Covariates are: indicators for ponds 2 to 8 (P2 - P8) with P1 as the reference level, and DNA collection method, (Glass Microfiber (GF) and Ethanol (E)) with Cellulose as the reference level

Figure 7b shows that, using pond 1 as a reference level, ponds 4 and 7 have a negative impact on DNA collection. For the DNA availability covariates shown in Figure 7a and for the other pond effects and DNA collection methods shown in Figure 7b, the inclusion of zero within the 95% PCIs of coefficients suggests the data do not provide strong evidence that these covariates have a non-zero effect.

**Fig. 8:**
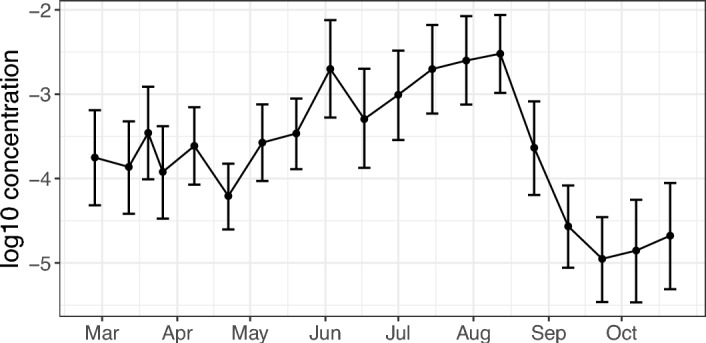
Great crested newts: Posterior means (circles) and 95% PCIs (bars) of the $\hbox {log}_{10}$-DNA ($\mu$g/mL) concentrations between February 2015 and October 2015

We can see the results of the amount of log-DNA available at the site in Figure 8 (we use base 10 here for comparison with Buxton et al. [6]). DNA concentration increases over the summer months and then quickly decreases over autumn, concurrent with larval metamorphosis and emergence from the ponds into the terrestrial environment.

Table 5 shows the posterior means and 95% PCIs for the CT variance parameters, $a_1$ and $a_2$, the probabilities of contamination and inhibition, $p_c$ and $p_h$, the DNA availability AR(1) term $\rho$, and the DNA availability and collection standard deviations, $\tau$, $\tau _1,$ and $\sigma$. The posterior means for $p_c$ (0.006 [0.003, 0.011]) and $p_h$ (0.031 [0.019, 0.044]) are very small, and are similar to the simulation parameters in which $(p_c, p_h) = (0.01, 0.02)$. Table 1 shows that the effect of ignoring contamination, inhibition, or heteroscedasticity leads to an increase in MSE and a reduction in nominal coverage, particularly when *M* and *K* were large.

**Table 5:** Great crested newts: Posterior means and 95% PCIs of variance parameters, $a_1, a_2$, probabilities of contamination and inhibition $p_c, p_h$, DNA availability AR(1) terms $\rho$, and DNA availability and collection standard deviations $\tau$, $\tau _1$, and $\sigma .$

Parameter	Mean	95% PCI
$a_1$	-3.046	(-3.249, -2.833)
$a_2$	-0.316	(-0.341, -0.290)
$p_c$	0.006	(0.003, 0.011)
$p_h$	0.031	(0.019, 0.044)
$\rho$	1.009	(0.934, 1.080)
$\tau$	1.251	(0.817, 1.876)
$\tau _1$	0.886	(0.421, 2.048)
$\sigma$	1.332	(1.186, 1.499)

## Discussion

qPCR methods are widely used for monitoring species distributions. This paper provides a statistical framework for the corresponding data, linking CT values to the concentration of species’ DNA in the environment across time and space whilst accounting for covariates. The model allows for contamination and inhibition of replicates during the PCR stage, and accounts for heteroscedasticity in CT values over log-DNA concentration. This is a unifying framework, propagating uncertainty through all stages of analysis, DNA availability, collection, and PCR analysis. Whilst analyses can be conducted on the back-transformed CT values directly [6], these often fail to account for the full data-generating process (see Section S3 for comparison with linear mixed effects models). We use extensive simulation studies, under different survey designs, to show that ignoring contamination and inhibition can lead to biased inferences about DNA concentration, and that ignoring CT heteroscedasticity leads to over-confident inferences that do not have the nominal coverage. These simulations also highlight the need for replication at both the sample and replicate level [7]. We apply the model to three case studies, one for a protected species and two for an invasive species.

Our model relies on a number of justifiable, and in some cases necessary, assumptions. Specifically, we assume that replicates from the same sample contain the same concentration of DNA; any variation between replicates in reality is then absorbed into the variation of CT values. Sharing the CT variation regression coefficients across plates helps to borrow information, though this assumption could be relaxed to model plate effects in a hierarchical model (as we do for the CT mean regression coefficients). We assume that no replicate can be simultaneously contaminated and inhibited, and this assumption is necessary for the model to be identifiable. Currently we also only allow for contamination or inhibition in individual replicates (rather than at the sample level), where contaminated or inhibited replicates are identified by being outliers with respect to other CT values associated with that sample. The other potential source of contamination in these studies is during the collection of samples at the site [3], or during the processing of samples [8, 23]. Unless samples are taken from the same location at the same time, or there are grounds for sensible assumptions about the distribution of (log-)DNA across a site, it may be difficult to identify contaminated samples under this method (as samples with vastly different amounts of log-DNA may be due to some un-modelled heterogeneity in the distribution of log-DNA across the site). Because we do not model contamination or inhibition at the collection stage, we rely on the noise term $\sigma ^2$ to capture any resulting shifts in the CT values associated with that sample. However this modelling framework relies on good practice in the field for that to be a reasonable assumption. Large amounts of contamination or inhibition would lead to significant shifts in CT values and $\sigma ^2$ would be insufficient to cover the increased uncertainty. For the case study of zebra mussels at a single time point, during sampling at each site, negative field controls were taken and analysed to test for contamination at the collection stage. For six sites, the negative field controls contained replicates that amplified, indicating potential contamination of all samples collected at those sites. Our model does not account for this, however the amount of contamination in the negative field controls was negligible when compared to the amount of DNA in the collected samples. For very small amounts of contamination at this stage, the noise term for DNA collection $\sigma ^2$ is thought to be enough to account for this presence of additional DNA. A simulation study of the effect of unaccounted sample contamination and inhibition can be found in Section S5. Tables S21 and S22 show that posterior distributions for $l_{i,t}$ and covariate coefficients ${\varvec{\beta }}_b$ and ${\varvec{\beta }}_w$ are fairly robust to small levels of inhibition and contamination at the collection stage, but that bias and uncertainty increase as these levels increase. To relax these assumptions, the inclusion of results from negative field controls in the model could help us extend the model to account for more sources of contamination, as the probability and distribution of contamination during the sample collection stage would be better understood. However, negative field controls are not always conducted: the zebra mussels case studies both used negative field controls (only the single time point survey indicated contamination), but the great crested newt study did not.

Our model takes the concentration of DNA $l_{i,t}$ to be constant throughout the site. The amount of DNA in the collected sample, $v_{imt}$, is conditional on $l_{i,t}$, and stochasticity can be explained through the noise term $\sigma ^2$ or sample-specific covariates. If however $l_{i,t}$ varies significantly throughout the site, and in particular over the sampling locations, then this variation will appear through the noise term $\sigma ^2$. If the variation of DNA concentration throughout the site can be explained by covariates, and these are included in the model for DNA collection, then the ${\varvec{\beta }}_w$ coefficients will indicate that the DNA in samples varies according to those covariates. Therefore in the case where $l_{i,t}$ varies considerably throughout the site, then inferred $l_{i,t}$ values can be interpreted as the average DNA concentration across sampling locations at the site and over the sampling covariates. For example, in the great crested newt case study (Section 4.3), we used pond as an effect on collection rate, and inferred that DNA in collected samples varied by pond number. However, if DNA concentrations in each pond differ, then these pond coefficients may in fact be indicating heterogeneity in DNA across the site, rather than an effect on collection rate. Currently, this model cannot differentiate between these two sources of variation in DNA concentration in samples.

In our simulation study, the mean concentration of contaminant $\lambda$ was such that it would not be detectable across all contaminated replicates. This is because the amount of contamination is only relevant depending on the amount of DNA already in the sample. For example a small concentration of contaminant DNA in a replicate that already has a high concentration of DNA is not going to significantly affect the CT values associated with that replicate. This was chosen to more closely mimic how contamination may affect analysis in real-world applications - where contamination is primarily a concern for low DNA concentration samples rather than abundant samples. Similarly, inhibition was modelled in such a way as to delay the amplification process proportionately to the amount of DNA in the sample, so that inhibition is more detectable in DNA abundant samples compared to DNA sparse samples. Despite the small underestimation of probabilities of contamination and inhibition in the model, our simulation study shows that not accounting for these processes leads to worse outcomes.

This model only estimates the amount of DNA present in the environment at time of sampling, rather than an estimate of species abundance at a site. Linking inferred DNA concentration to species abundance would require knowledge about DNA shedding rates, estimates of how long DNA persists in the environment, the effects of environmental factors, species habitat use, etc. These would then need expert knowledge to interpret how DNA availability links back to changes in species abundance at a site. Species detection/non-detection or counts can be integrated into this model in order to investigate the relationship between DNA concentration in the environment and species abundance. For example, Buxton et al. [6] show that contributions to DNA in the ponds vary seasonally with the different life stages of great crested newts. During the breeding season, the adult population changes very little, but DNA concentration increases due to breeding activities. Post breeding activity, DNA concentration increases as larval abundance increases, but the adult population decreases as individuals return to land. Therefore linking DNA concentration to adult population abundance is complicated by the seasonal behaviour of the species.

We had a small number of covariates available in our case studies, and a subset of the covariates that were not strongly correlated with each other were included in the model in each case study, but no formal model selection was carried out. In case studies with large numbers of covariates, covariate choice and combinations may be best implemented within a Bayesian variable selection approach [17]. We also assume that log-DNA concentrations are a linear function of covariates, but could consider extensions to more flexible models or interactions subject to data availability and quality. The model currently treats $\beta _{b,0}$ as an initial condition rather than a long term mean value, meaning that log-DNA concentrations over time are primarily determined by covariates. We also have not enforced $|\rho | < 1$, and do not assume stationarity, as eDNA can rapidly accumulate or decay under certain conditions. In our case studies, posterior means of $\rho$ are close to 1, indicating strong temporal dependence. Alternative parametrisations for the temporal model could incorporate an explicit intercept at each time step, imposing a long term mean structure on log-DNA concentrations. This could be useful if prior knowledge suggests DNA levels fluctuate around some baseline rather than being driven by covariates. Additionally, enforcing stationarity may be appropriate in cases where long term persistence is expected.

Our case studies also each only considered a small number of sites, and so our model did not consider any spatial correlation between the sampled sites, and instead only focused on accounting for fixed effects. qPCR methods are used on a much larger scale (Buxton et al. [2] analyses qPCR results for great crested newts on a national scale in England), where more sophisticated spatial models may be considered for the DNA availability stage.

Our simulation study considered a wide range of survey designs (different levels of replication in M and K), three models (where one model ignores CT heteroscedasticity and one ignores contamination and inhibition), and two levels of contamination and inhibition probabilities (one high and one low). Further simulations could be considered to compare the effects of increasing or decreasing noise at all stages of the analysis, or by increasing the slope of the log-variance of CT values to see if this increases the bias of results from Model 2. We could also vary the concentration of the contaminant DNA, or change the effect of inhibition to see how these affect the model’s ability to detect these affected replicates.

This paper provides a general framework for inferring DNA concentration and quantifying covariate effects whilst accounting for both potential contamination or inhibition of replicates and the heteroscedasticity in CT values obtained from qPCR analyses. Simulation results highlight the importance of replication in both the number of samples *M* and the number of PCR replicates *K*. qPCR continues to be the preferred monitoring tool for single species monitoring, being used for large scale monitoring of elusive species such as great crested newts [33], invasive species such as zebra mussels [1], and even for investigating ancient DNA spanning centuries [24].

## Supplementary information

Supplementary material gives additional information on prior distributions and posterior summaries for simulations and all case studies presented in this paper. It also contains a comparison of Model 1 to a linear mixed effects model, a prior sensitivity analysis, and a simulation study with sample contamination.

## Supplementary Information

Below is the link to the electronic supplementary material.

Supplementary file 1 (pdf 547 KB)
