# Supplementary material for: More than presence-absence; modelling (e)DNA concentration across time and space from qPCR survey data
Source: J Stat Theory Pract. 2025 Aug 5;19(4):68. doi: 10.1007/s42519-025-00477-9 (PMC12325409; doi:10.1007/s42519-025-00477-9)
Supplement: Supplementary file 1 — (pdf 547 KB) [file 42519_2025_477_MOESM1_ESM.pdf]

Supplementary material for the article "More than presence-absence; modelling (e)DNA concentration across time and space from qPCR survey data"

Milly Jones[1*], Eleni Matechou[1], Diana Cole[1], Alex Diana[2], Jim Griffin[3], Sara Peixoto[4], Lori Lawson Handley[5], Andrew Buxton[6]

[1*]School of Mathematics, Statistics, and Actuarial Science, University of Kent, Cornwallis South, Canterbury, Kent, CT2 7NF.
[2]School of Mathematics, Statistics, and Actuarial Science, University of Essex, Wivenhoe Park, Colchester, Essex, CO4 3SQ.
[3]Department of Statistical Science, University College London, Gower Street, London, WC1E 6BT.
[4]School of Natural Sciences, University of Hull, Hull, HU6 7RX.
[5]Lake Ecosystems Group, UK Centre for Ecology and Hydrology, Lancaster Environment Centre, Bailrigg, Lancaster, LA1 4AP.
[6]NatureSpace, 22 St Peter's Street, Stamford Lincolnshire, PE9 2PF.

*Corresponding author(s). E-mail(s): mlj23@kent.ac.uk;
Contributing authors: e.matechou@kent.ac.uk; d.j.cole@kent.ac.uk; ad23269@essex.ac.uk; j.griffin@ucl.ac.uk; sara.peixoto15@gmail.com; lorlaw@ceh.ac.uk; andy.buxton@naturespaceuk.com;

Supplementary material for the article in the Journal of Statistical Theory and Practice

S1 Simulation studies

Each MCMC was run over 1 chain. The chains were run for 11000 iterations, with no thinning and a 1000 iteration burn-in for 10000 total samples. Table S1 shows the prior distributions used in each of the simulations. Throughout, the Inverse Gamma distribution uses the shape/scale parametrisation. See Section S4 for a prior sensitivity analysis of the plate regression parameters $\alpha_p^1, \alpha_p^2, a_1$ and a_2, and for an alternative prior specification for $\beta_{b,0}$.

Table S1: Table of prior distributions used in the simulations of Section 3. Columns indicate the parameter name, its use in the model, and the prior distribution used.

Parameter	Use	Prior
$\boldsymbol{\beta}_b$	Coefficients on site covariates	$\sim \text{N}(0, 1)$
$\boldsymbol{\beta}_w$	Coefficients on sample covariates	$\sim \text{N}(0, 1)$
$\beta_{b,0}$	Intercept on log-DNA at $t = 1$	$\sim \text{N}(6, 1)$
τ^2	Variance of log-DNA across time	$\sim \text{InvGamma}(2, 1)$
τ_1^2	Variance of log-DNA across sites	$\sim \text{InvGamma}(2, 1)$
σ^2	Variance of log-DNA across samples	$\sim \text{InvGamma}(2, 1)$
$(p_c, 1 - p_c - p_h, p_h)$	Probability of contamination or inhibition	$\sim \text{Dirichlet}(0.01, 0.98, 0.01)$
a_1	CT log-variance intercept term	$\sim \text{N}(0.2, 1)$
a_2	CT log-variance slope term	$\sim \text{N}(-0.25, 1)$
α_p^1	Plate regression intercepts	$\sim \text{N}(44, 1)$
α_p^2	Plate regression slopes	$\sim \text{N}(-1.7, 1)$
ρ_0	AR(1) coefficient mean	$\sim \text{N}(1, 1)$
σ_ρ^2	AR(1) coefficient noise	$\sim \text{InvGamma}(2, .1)$
σ_c	Standard deviation for contaminated or inhibitied replicate	30
CT.max	Maximum CT value	40

For Model 2, where we are using constant plate variance, σ_p^2, we use the following prior distribution:

$$\sigma_p^2 \sim \text{InvGamma}(2, 0.5) \qquad \text{for } p = 1, \cdots, P,$$

where P is the maximum number of plates.

S1.1 Other Parameter Results

In this section we present posterior summaries for other model parameters in the simulations. Each table shows the mean bias (MB), mean width of the 95% PCIs (R), and the mean proportion of intervals containing zero (Z) across the $N = 100$ simulations. Tables S2 and S3 give results for the covariate coefficients $\boldsymbol{\beta}_b$ and $\boldsymbol{\beta}_w$ under $(p_c, p_h) = (0.05, 0.1)$ and $(p_c, p_h) = (0.01, 0.02)$ respectively. Tables S4 and S5 give results for the variance parameters a_1 and a_2 and the probabilities of contamination and inhibition p_c and p_h under $(p_c, p_h) = (0.05, 0.1)$ and $(p_c, p_h) = (0.01, 0.02)$ respectively.

Table S2: Mean bias (MB), the mean width of the 95% PCI (R), and the mean number of intervals containing zero (Z) of the covariate coefficients $\boldsymbol{\beta}_b, \boldsymbol{\beta}_w$ across Model 1 (full model), Model 2 (constant CT variance), and Model 3 (ignoring contamination and inhibition) under different sampling designs. $\boldsymbol{\beta}_b = (1, -1)$, $\boldsymbol{\beta}_w = (1, -1)$, and the probability of contamination and inhibition $(p_c, p_h) = (0.05, 0.1)$.

	Model 1			Model 2			Model 3		
M = 1	MB	R	Z	MB	R	Z	MB	R	Z
K = 1									
$\boldsymbol{\beta}_b[1]$	-0.022	0.545	0.000	-0.020	0.536	0.000	-0.034	0.590	0.000
$\boldsymbol{\beta}_b[2]$	0.156	1.043	0.080	0.154	1.021	0.080	0.201	1.123	0.200
$\boldsymbol{\beta}_w[1]$	-0.031	0.550	0.000	-0.028	0.543	0.000	-0.042	0.592	0.000
$\boldsymbol{\beta}_w[2]$	0.123	1.043	0.130	0.125	1.026	0.120	0.160	1.125	0.200
K = 2									
$\boldsymbol{\beta}_b[1]$	-0.014	0.478	0.000	-0.023	0.461	0.000	-0.056	0.505	0.000
$\boldsymbol{\beta}_b[2]$	0.091	0.909	0.020	0.093	0.882	0.010	0.145	0.967	0.080
$\boldsymbol{\beta}_w[1]$	-0.015	0.475	0.000	-0.018	0.459	0.000	-0.060	0.506	0.000
$\boldsymbol{\beta}_w[2]$	0.048	0.908	0.020	0.062	0.871	0.020	0.112	0.966	0.080
K = 5									
$\boldsymbol{\beta}_b[1]$	-0.007	0.427	0.000	-0.035	0.415	0.000	-0.038	0.457	0.000
$\boldsymbol{\beta}_b[2]$	0.055	0.820	0.010	0.085	0.795	0.010	0.125	0.883	0.070
$\boldsymbol{\beta}_w[1]$	-0.009	0.424	0.000	-0.034	0.412	0.000	-0.052	0.455	0.000
$\boldsymbol{\beta}_w[2]$	0.097	0.820	0.020	0.124	0.796	0.020	0.149	0.880	0.050
K = 10									
$\boldsymbol{\beta}_b[1]$	0.004	0.424	0.000	-0.032	0.408	0.000	-0.021	0.454	0.000
$\boldsymbol{\beta}_b[2]$	0.043	0.813	0.010	0.082	0.783	0.010	0.093	0.865	0.030
$\boldsymbol{\beta}_w[1]$	0.006	0.423	0.000	-0.033	0.404	0.000	-0.012	0.454	0.000
$\boldsymbol{\beta}_w[2]$	0.071	0.812	0.000	0.103	0.781	0.000	0.103	0.866	0.010
M = 2	MB	R	Z	MB	R	Z	MB	R	Z
K = 1									
$\boldsymbol{\beta}_b[1]$	-0.002	0.409	0.000	-0.006	0.405	0.000	-0.013	0.441	0.000
$\boldsymbol{\beta}_b[2]$	0.105	0.792	0.010	0.112	0.780	0.010	0.132	0.854	0.040
$\boldsymbol{\beta}_w[1]$	-0.035	0.346	0.000	-0.038	0.343	0.000	-0.049	0.378	0.000
$\boldsymbol{\beta}_w[2]$	0.055	0.669	0.000	0.053	0.661	0.000	0.076	0.730	0.000
K = 2									
$\boldsymbol{\beta}_b[1]$	-0.001	0.369	0.000	-0.016	0.359	0.000	-0.042	0.397	0.000
$\boldsymbol{\beta}_b[2]$	0.071	0.710	0.000	0.082	0.689	0.000	0.118	0.763	0.010
$\boldsymbol{\beta}_w[1]$	0.010	0.292	0.000	-0.007	0.285	0.000	-0.030	0.324	0.000
$\boldsymbol{\beta}_w[2]$	0.045	0.569	0.000	0.050	0.550	0.000	0.090	0.629	0.000
K = 5									
$\boldsymbol{\beta}_b[1]$	0.007	0.340	0.000	-0.024	0.332	0.000	-0.020	0.363	0.000
$\boldsymbol{\beta}_b[2]$	0.041	0.659	0.000	0.071	0.637	0.000	0.068	0.702	0.000
$\boldsymbol{\beta}_w[1]$	0.005	0.262	0.000	-0.020	0.255	0.000	-0.020	0.285	0.000
$\boldsymbol{\beta}_w[2]$	0.004	0.509	0.000	0.031	0.496	0.000	0.037	0.557	0.000
K = 10									

Continued on next page

	Model 1			Model 2			Model 3		
$\boldsymbol{\beta}_b[1]$	0.000	0.336	0.000	-0.042	0.321	0.000	0.024	0.379	0.000
$\boldsymbol{\beta}_b[2]$	0.055	0.652	0.000	0.089	0.629	0.000	0.052	0.735	0.010
$\boldsymbol{\beta}_w[1]$	0.003	0.261	0.000	-0.030	0.251	0.000	0.010	0.284	0.000
$\boldsymbol{\beta}_w[2]$	-0.014	0.508	0.000	0.024	0.490	0.000	-0.006	0.555	0.000
M = 5	MB	R	Z	MB	R	Z	MB	R	Z
K = 1									
$\boldsymbol{\beta}_b[1]$	-0.016	0.309	0.000	-0.020	0.304	0.000	-0.047	0.338	0.000
$\boldsymbol{\beta}_b[2]$	0.046	0.608	0.000	0.050	0.601	0.000	0.097	0.662	0.010
$\boldsymbol{\beta}_w[1]$	-0.008	0.203	0.000	-0.011	0.200	0.000	-0.041	0.230	0.000
$\boldsymbol{\beta}_w[2]$	0.025	0.394	0.000	0.030	0.391	0.000	0.069	0.450	0.000
K = 2									
$\boldsymbol{\beta}_b[1]$	0.009	0.287	0.000	-0.011	0.279	0.000	-0.025	0.306	0.000
$\boldsymbol{\beta}_b[2]$	-0.005	0.560	0.000	0.019	0.545	0.000	0.050	0.598	0.000
$\boldsymbol{\beta}_w[1]$	0.003	0.169	0.000	-0.015	0.166	0.000	-0.025	0.188	0.000
$\boldsymbol{\beta}_w[2]$	0.008	0.331	0.000	0.028	0.320	0.000	0.050	0.368	0.000
K = 5									
$\boldsymbol{\beta}_b[1]$	0.005	0.277	0.000	-0.026	0.269	0.000	0.026	0.308	0.000
$\boldsymbol{\beta}_b[2]$	0.011	0.535	0.000	0.041	0.521	0.000	0.001	0.593	0.000
$\boldsymbol{\beta}_w[1]$	0.008	0.152	0.000	-0.015	0.150	0.000	0.020	0.170	0.000
$\boldsymbol{\beta}_w[2]$	-0.011	0.298	0.000	0.015	0.291	0.000	-0.015	0.332	0.000
K = 10									
$\boldsymbol{\beta}_b[1]$	0.007	0.272	0.000	-0.035	0.261	0.000	0.117	0.382	0.000
$\boldsymbol{\beta}_b[2]$	0.007	0.529	0.000	0.045	0.506	0.000	-0.055	0.719	0.000
$\boldsymbol{\beta}_w[1]$	0.009	0.148	0.000	-0.019	0.144	0.000	0.054	0.165	0.000
$\boldsymbol{\beta}_w[2]$	0.002	0.290	0.000	0.032	0.283	0.000	-0.032	0.325	0.000
M = 10	MB	R	Z	MB	R	Z	MB	R	Z
K = 1									
$\boldsymbol{\beta}_b[1]$	-0.002	0.270	0.000	-0.009	0.266	0.000	-0.052	0.293	0.000
$\boldsymbol{\beta}_b[2]$	0.016	0.526	0.000	0.026	0.517	0.000	0.070	0.570	0.000
$\boldsymbol{\beta}_w[1]$	-0.005	0.138	0.000	-0.009	0.135	0.000	-0.039	0.159	0.000
$\boldsymbol{\beta}_w[2]$	0.014	0.269	0.000	0.018	0.267	0.000	0.055	0.311	0.000
K = 2									
$\boldsymbol{\beta}_b[1]$	0.016	0.251	0.000	-0.006	0.245	0.000	-0.001	0.267	0.000
$\boldsymbol{\beta}_b[2]$	0.007	0.496	0.000	0.028	0.483	0.000	0.024	0.528	0.000
$\boldsymbol{\beta}_w[1]$	0.003	0.114	0.000	-0.014	0.111	0.000	-0.010	0.127	0.000
$\boldsymbol{\beta}_w[2]$	-0.010	0.222	0.000	0.011	0.217	0.000	0.005	0.249	0.000
K = 5									
$\boldsymbol{\beta}_b[1]$	0.013	0.245	0.000	-0.021	0.239	0.000	0.107	0.333	0.000
$\boldsymbol{\beta}_b[2]$	-0.000	0.480	0.000	0.037	0.469	0.000	-0.072	0.646	0.000
$\boldsymbol{\beta}_w[1]$	0.014	0.104	0.000	-0.009	0.102	0.000	0.044	0.118	0.000
$\boldsymbol{\beta}_w[2]$	-0.008	0.204	0.000	0.014	0.199	0.000	-0.038	0.230	0.000
K = 10									
$\boldsymbol{\beta}_b[1]$	0.007	0.246	0.000	-0.036	0.236	0.000	0.270	0.495	0.000

Continued on next page

	Model 1			Model 2			Model 3		
$\boldsymbol{\beta}_b[2]$	0.012	0.482	0.000	0.056	0.464	0.000	-0.183	0.921	0.010
$\boldsymbol{\beta}_w[1]$	0.005	0.103	0.000	-0.022	0.101	0.000	0.078	0.120	0.000
$\boldsymbol{\beta}_w[2]$	-0.007	0.201	0.000	0.019	0.196	0.000	-0.077	0.232	0.000

Table S3: Mean bias (MB), the mean width of the 95% PCI (R), and the mean number of intervals containing zero (Z) of the covariate coefficients $\boldsymbol{\beta}_b, \boldsymbol{\beta}_w$ across Model 1 (full model), Model 2 (constant CT variance), and Model 3 (ignoring contamination and inhibition) under different sampling designs. $\boldsymbol{\beta}_b = (1, -1)$, $\boldsymbol{\beta}_w = (1, -1)$, and the probability of contamination and inhibition $(p_c, p_h) = (0.01, 0.02)$.

	Model 1			Model 2			Model 3		
M = 1	MB	R	Z	MB	R	Z	MB	R	Z
K = 1									
$\boldsymbol{\beta}_b[1]$	-0.015	0.457	0.000	-0.020	0.455	0.000	-0.017	0.468	0.000
$\boldsymbol{\beta}_b[2]$	0.101	0.884	0.050	0.103	0.880	0.040	0.107	0.908	0.040
$\boldsymbol{\beta}_w[1]$	0.000	0.464	0.000	-0.003	0.459	0.000	0.003	0.477	0.000
$\boldsymbol{\beta}_w[2]$	0.081	0.887	0.030	0.087	0.883	0.030	0.091	0.904	0.060
K = 2									
$\boldsymbol{\beta}_b[1]$	-0.011	0.439	0.000	-0.024	0.431	0.000	-0.024	0.450	0.000
$\boldsymbol{\beta}_b[2]$	0.079	0.843	0.010	0.094	0.829	0.010	0.099	0.867	0.030
$\boldsymbol{\beta}_w[1]$	-0.008	0.442	0.000	-0.022	0.437	0.000	-0.019	0.455	0.000
$\boldsymbol{\beta}_w[2]$	0.075	0.841	0.010	0.088	0.826	0.010	0.081	0.864	0.010
K = 5									
$\boldsymbol{\beta}_b[1]$	-0.010	0.415	0.000	-0.035	0.405	0.000	-0.024	0.423	0.000
$\boldsymbol{\beta}_b[2]$	0.112	0.798	0.000	0.133	0.782	0.000	0.138	0.814	0.000
$\boldsymbol{\beta}_w[1]$	-0.010	0.422	0.000	-0.033	0.412	0.000	-0.022	0.429	0.000
$\boldsymbol{\beta}_w[2]$	0.073	0.806	0.010	0.097	0.790	0.000	0.098	0.817	0.020
K = 10									
$\boldsymbol{\beta}_b[1]$	0.007	0.413	0.000	-0.031	0.399	0.000	-0.009	0.419	0.000
$\boldsymbol{\beta}_b[2]$	0.059	0.793	0.010	0.092	0.765	0.010	0.074	0.809	0.010
$\boldsymbol{\beta}_w[1]$	0.001	0.413	0.000	-0.034	0.399	0.000	-0.010	0.420	0.000
$\boldsymbol{\beta}_w[2]$	0.086	0.793	0.000	0.120	0.768	0.000	0.096	0.810	0.010
M = 2	MB	R	Z	MB	R	Z	MB	R	Z
K = 1									
$\boldsymbol{\beta}_b[1]$	0.003	0.364	0.000	-0.005	0.361	0.000	-0.007	0.376	0.000
$\boldsymbol{\beta}_b[2]$	0.049	0.697	0.000	0.055	0.691	0.000	0.062	0.721	0.000
$\boldsymbol{\beta}_w[1]$	-0.005	0.288	0.000	-0.008	0.287	0.000	-0.013	0.299	0.000
$\boldsymbol{\beta}_w[2]$	0.031	0.559	0.000	0.037	0.556	0.000	0.039	0.583	0.000
K = 2									
$\boldsymbol{\beta}_b[1]$	0.010	0.341	0.000	-0.007	0.336	0.000	-0.003	0.351	0.000
$\boldsymbol{\beta}_b[2]$	0.060	0.672	0.000	0.078	0.660	0.000	0.081	0.691	0.000
$\boldsymbol{\beta}_w[1]$	-0.022	0.272	0.000	-0.040	0.265	0.000	-0.034	0.281	0.000

Continued on next page

	Model 1			Model 2			Model 3		
$\boldsymbol{\beta}_w[2]$	0.041	0.528	0.000	0.057	0.517	0.000	0.066	0.545	0.000
K = 5									
$\boldsymbol{\beta}_b[1]$	0.005	0.338	0.000	-0.022	0.327	0.000	-0.006	0.348	0.000
$\boldsymbol{\beta}_b[2]$	0.051	0.652	0.000	0.078	0.633	0.000	0.066	0.667	0.000
$\boldsymbol{\beta}_w[1]$	-0.009	0.259	0.000	-0.034	0.252	0.000	-0.024	0.266	0.000
$\boldsymbol{\beta}_w[2]$	0.014	0.510	0.000	0.039	0.497	0.000	0.031	0.526	0.000
K = 10									
$\boldsymbol{\beta}_b[1]$	-0.017	0.330	0.000	-0.052	0.320	0.000	-0.023	0.340	0.000
$\boldsymbol{\beta}_b[2]$	0.034	0.645	0.000	0.072	0.620	0.000	0.039	0.667	0.000
$\boldsymbol{\beta}_w[1]$	-0.005	0.255	0.000	-0.037	0.247	0.000	-0.016	0.263	0.000
$\boldsymbol{\beta}_w[2]$	0.028	0.501	0.000	0.061	0.484	0.000	0.039	0.515	0.000
M = 5	MB	R	Z	MB	R	Z	MB	R	Z
K = 1									
$\boldsymbol{\beta}_b[1]$	-0.006	0.283	0.000	-0.015	0.280	0.000	-0.020	0.293	0.000
$\boldsymbol{\beta}_b[2]$	0.050	0.553	0.000	0.059	0.550	0.000	0.070	0.572	0.000
$\boldsymbol{\beta}_w[1]$	0.003	0.167	0.000	-0.004	0.166	0.000	-0.006	0.176	0.000
$\boldsymbol{\beta}_w[2]$	0.005	0.325	0.000	0.010	0.323	0.000	0.019	0.343	0.000
K = 2									
$\boldsymbol{\beta}_b[1]$	-0.000	0.279	0.000	-0.019	0.273	0.000	-0.015	0.286	0.000
$\boldsymbol{\beta}_b[2]$	0.041	0.542	0.000	0.066	0.533	0.000	0.065	0.556	0.000
$\boldsymbol{\beta}_w[1]$	0.006	0.158	0.000	-0.011	0.154	0.000	-0.007	0.164	0.000
$\boldsymbol{\beta}_w[2]$	0.004	0.308	0.000	0.020	0.301	0.000	0.022	0.322	0.000
K = 5									
$\boldsymbol{\beta}_b[1]$	0.003	0.270	0.000	-0.023	0.263	0.000	-0.003	0.282	0.000
$\boldsymbol{\beta}_b[2]$	0.038	0.526	0.000	0.063	0.510	0.000	0.047	0.548	0.000
$\boldsymbol{\beta}_w[1]$	0.003	0.149	0.000	-0.019	0.147	0.000	-0.001	0.154	0.000
$\boldsymbol{\beta}_w[2]$	-0.003	0.292	0.000	0.018	0.285	0.000	0.002	0.301	0.000
K = 10									
$\boldsymbol{\beta}_b[1]$	-0.001	0.267	0.000	-0.044	0.258	0.000	0.030	0.313	0.000
$\boldsymbol{\beta}_b[2]$	0.051	0.523	0.000	0.088	0.503	0.000	0.038	0.594	0.000
$\boldsymbol{\beta}_w[1]$	0.001	0.148	0.000	-0.029	0.146	0.000	0.009	0.156	0.000
$\boldsymbol{\beta}_w[2]$	-0.006	0.290	0.000	0.024	0.282	0.000	-0.016	0.302	0.000
M = 10	MB	R	Z	MB	R	Z	MB	R	Z
K = 1									
$\boldsymbol{\beta}_b[1]$	-0.005	0.253	0.000	-0.014	0.251	0.000	-0.023	0.264	0.000
$\boldsymbol{\beta}_b[2]$	0.038	0.497	0.000	0.048	0.492	0.000	0.062	0.517	0.000
$\boldsymbol{\beta}_w[1]$	-0.001	0.114	0.000	-0.007	0.113	0.000	-0.012	0.121	0.000
$\boldsymbol{\beta}_w[2]$	0.005	0.222	0.000	0.011	0.221	0.000	0.014	0.236	0.000
K = 2									
$\boldsymbol{\beta}_b[1]$	-0.001	0.246	0.000	-0.020	0.242	0.000	-0.014	0.253	0.000
$\boldsymbol{\beta}_b[2]$	0.020	0.482	0.000	0.040	0.472	0.000	0.033	0.496	0.000
$\boldsymbol{\beta}_w[1]$	-0.002	0.106	0.000	-0.019	0.104	0.000	-0.011	0.111	0.000
$\boldsymbol{\beta}_w[2]$	-0.011	0.207	0.000	0.006	0.204	0.000	0.000	0.217	0.000

Continued on next page

	Model 1			Model 2			Model 3		
K = 5									
$\boldsymbol{\beta}_b[1]$	-0.005	0.246	0.000	-0.036	0.240	0.000	0.009	0.272	0.000
$\boldsymbol{\beta}_b[2]$	0.029	0.482	0.000	0.061	0.468	0.000	0.017	0.526	0.000
$\boldsymbol{\beta}_w[1]$	0.003	0.103	0.000	-0.018	0.101	0.000	0.005	0.107	0.000
$\boldsymbol{\beta}_w[2]$	-0.010	0.200	0.000	0.012	0.196	0.000	-0.011	0.209	0.000
K = 10									
$\boldsymbol{\beta}_b[1]$	-0.006	0.243	0.000	-0.045	0.235	0.000	0.100	0.390	0.000
$\boldsymbol{\beta}_b[2]$	-0.001	0.476	0.000	0.047	0.461	0.000	-0.062	0.727	0.030
$\boldsymbol{\beta}_w[1]$	0.002	0.101	0.000	-0.024	0.100	0.000	0.026	0.108	0.000
$\boldsymbol{\beta}_w[2]$	-0.003	0.197	0.000	0.024	0.193	0.000	-0.029	0.209	0.000

Table S4: Mean bias (MB), the mean width of the 95% PCIs (R), and the mean proportion of intervals containing zero (Z) of parameters a_1, a_2, p_c and p_h across Model 1 (full model), Model 2 (constant CT variance), and Model 3 (ignoring contamination and inhibition) under different sampling designs. Variance parameters $(a_1, a_2) = (0.2, -0.25)$, and probability of contamination and inhibition $(p_c, p_h) = (0.05, 0.1)$.

	Model 1			Model 2			Model 3		
M = 1	MB	R	Z	MB	R	Z	MB	R	Z
K = 1									
a_1	0.114	0.267	0.000	-	-	-	-3.032	0.423	0.000
a_2	-0.003	0.026	0.010	-	-	-	0.402	0.037	0.000
p_c	-0.034	0.008	0.000	-0.034	0.008	0.000	-	-	-
p_h	-0.001	0.019	0.000	0.001	0.019	0.000	-	-	-
K = 2									
a_1	0.130	0.261	0.000	-	-	-	-3.201	0.412	0.000
a_2	-0.006	0.023	0.010	-	-	-	0.415	0.037	0.000
p_c	-0.033	0.008	0.000	-0.032	0.008	0.000	-	-	-
p_h	0.001	0.018	0.000	0.003	0.019	0.000	-	-	-
K = 5									
a_1	0.108	0.243	0.000	-	-	-	-3.555	0.374	0.000
a_2	-0.003	0.024	0.020	-	-	-	0.442	0.034	0.000
p_c	-0.030	0.008	0.000	-0.029	0.008	0.000	-	-	-
p_h	-0.000	0.018	0.000	0.002	0.018	0.000	-	-	-
K = 10									
a_1	0.088	0.217	0.000	-	-	-	-3.984	0.335	0.000
a_2	-0.001	0.023	0.010	-	-	-	0.480	0.031	0.000
p_c	-0.026	0.008	0.000	-0.026	0.008	0.000	-	-	-
p_h	-0.000	0.017	0.000	0.002	0.017	0.000	-	-	-
M = 2	MB	R	Z	MB	R	Z	MB	R	Z
K = 1									
a_1	0.114	0.265	0.010	-	-	-	-3.040	0.425	0.000

Continued on next page

	Model 1			Model 2			Model 3		
a_2	-0.005	0.023	0.000	-	-	-	0.403	0.037	0.000
p_c	-0.034	0.007	0.000	-0.034	0.008	0.000	-	-	-
p_h	-0.004	0.018	0.000	-0.002	0.018	0.000	-	-	-
K = 2									
a_1	0.142	0.259	0.000	-	-	-	-3.298	0.406	0.000
a_2	-0.001	0.023	0.010	-	-	-	0.421	0.036	0.000
p_c	-0.032	0.008	0.000	-0.031	0.008	0.000	-	-	-
p_h	-0.002	0.017	0.000	0.003	0.018	0.000	-	-	-
K = 5									
a_1	0.122	0.222	0.000	-	-	-	-3.824	0.341	0.000
a_2	-0.005	0.021	0.000	-	-	-	0.469	0.032	0.000
p_c	-0.027	0.008	0.000	-0.026	0.008	0.000	-	-	-
p_h	-0.002	0.016	0.000	0.000	0.017	0.000	-	-	-
K = 10									
a_1	0.074	0.187	0.000	-	-	-	-4.301	0.294	0.000
a_2	-0.003	0.020	0.000	-	-	-	0.513	0.029	0.000
p_c	-0.022	0.007	0.000	-0.022	0.008	0.000	-	-	-
p_h	-0.001	0.014	0.000	-0.001	0.015	0.000	-	-	-
M = 5	MB	R	Z	MB	R	Z	MB	R	Z
K = 1									
a_1	0.112	0.264	0.000	-	-	-	-3.097	0.444	0.000
a_2	-0.007	0.023	0.000	-	-	-	0.407	0.039	0.000
p_c	-0.034	0.007	0.000	-0.033	0.008	0.000	-	-	-
p_h	-0.008	0.017	0.000	-0.009	0.017	0.000	-	-	-
K = 2									
a_1	0.161	0.250	0.000	-	-	-	-3.719	0.389	0.000
a_2	-0.005	0.025	0.010	-	-	-	0.456	0.035	0.000
p_c	-0.029	0.008	0.000	-0.027	0.008	0.000	-	-	-
p_h	-0.005	0.017	0.000	-0.002	0.017	0.000	-	-	-
K = 5									
a_1	0.103	0.187	0.000	-	-	-	-4.365	0.281	0.000
a_2	-0.004	0.024	0.010	-	-	-	0.518	0.028	0.000
p_c	-0.021	0.007	0.000	-0.021	0.007	0.000	-	-	-
p_h	-0.003	0.016	0.000	-0.003	0.015	0.000	-	-	-
K = 10									
a_1	0.069	0.145	0.000	-	-	-	-4.662	0.240	0.000
a_2	-0.002	0.016	0.000	-	-	-	0.563	0.025	0.000
p_c	-0.017	0.006	0.000	-0.017	0.006	0.000	-	-	-
p_h	-0.003	0.012	0.000	-0.004	0.012	0.000	-	-	-
M = 10	MB	R	Z	MB	R	Z	MB	R	Z
K = 1									
a_1	0.118	0.266	0.010	-	-	-	-3.221	0.508	0.000
a_2	0.004	0.023	0.020	-	-	-	0.415	0.043	0.000

Continued on next page

	Model 1			Model 2			Model 3		
p_c	-0.033	0.007	0.000	-0.032	0.007	0.000	-	-	-
p_h	-0.019	0.015	0.000	-0.018	0.016	0.000	-	-	-
K = 2									
a_1	0.217	0.232	0.000	-	-	-	-4.174	0.349	0.000
a_2	-0.001	0.021	0.000	-	-	-	0.497	0.033	0.000
p_c	-0.025	0.007	0.000	-0.024	0.008	0.000	-	-	-
p_h	-0.010	0.014	0.000	-0.006	0.015	0.000	-	-	-
K = 5									
a_1	0.102	0.157	0.000	-	-	-	-4.778	0.258	0.000
a_2	-0.005	0.019	0.010	-	-	-	0.570	0.026	0.000
p_c	-0.016	0.006	0.000	-0.016	0.006	0.000	-	-	-
p_h	-0.004	0.013	0.000	-0.006	0.013	0.000	-	-	-
K = 10									
a_1	0.053	0.118	0.000	-	-	-	-4.895	0.206	0.000
a_2	-0.000	0.014	0.000	-	-	-	0.601	0.022	0.000
p_c	-0.014	0.005	0.000	-0.014	0.005	0.000	-	-	-
p_h	-0.004	0.011	0.000	-0.007	0.011	0.000	-	-	-

Table S5: Mean bias (MB), the mean width of the 95% PCIs (R), and the mean proportion of intervals containing zero (Z) of parameters a_1, a_2, p_c and p_h across Model 1 (full model), Model 2 (constant CT variance), and Model 3 (ignoring contamination and inhibition) under different sampling designs. Variance parameters $(a_1, a_2) = (0.2, -0.25)$, and probability of contamination and inhibition $(p_c, p_h) = (0.01, 0.02)$.

	Model 1			Model 2			Model 3		
M = 1	MB	R	Z	MB	R	Z	MB	R	Z
K = 1									
a_1	0.029	0.235	0.040	-	-	-	-0.760	0.424	0.190
a_2	-0.002	0.021	0.000	-	-	-	0.444	0.038	0.000
p_c	-0.007	0.003	0.000	-0.006	0.004	0.000	-	-	-
p_h	-0.000	0.009	0.000	0.002	0.009	0.000	-	-	-
K = 2									
a_1	0.038	0.229	0.060	-	-	-	-0.855	0.411	0.080
a_2	-0.003	0.021	0.000	-	-	-	0.454	0.037	0.000
p_c	-0.007	0.003	0.000	-0.006	0.004	0.000	-	-	-
p_h	-0.000	0.008	0.000	0.003	0.009	0.000	-	-	-
K = 5									
a_1	0.032	0.208	0.010	-	-	-	-1.069	0.370	0.030
a_2	-0.002	0.019	0.000	-	-	-	0.469	0.034	0.000
p_c	-0.006	0.003	0.000	-0.005	0.004	0.000	-	-	-
p_h	-0.000	0.008	0.000	0.002	0.009	0.000	-	-	-
K = 10									

Continued on next page

	Model 1			Model 2			Model 3		
a_1	0.019	0.185	0.000	-	-	-	-1.335	0.327	0.020
a_2	-0.001	0.018	0.000	-	-	-	0.492	0.031	0.000
p_c	-0.005	0.003	0.000	-0.005	0.004	0.000	-	-	-
p_h	-0.000	0.007	0.000	0.002	0.008	0.000	-	-	-
M = 2	MB	R	Z	MB	R	Z	MB	R	Z
K = 1									
a_1	0.012	0.234	0.060	-	-	-	-0.710	0.426	0.150
a_2	-0.001	0.021	0.000	-	-	-	0.442	0.038	0.000
p_c	-0.007	0.003	0.000	-0.006	0.004	0.000	-	-	-
p_h	-0.001	0.008	0.000	0.001	0.009	0.000	-	-	-
K = 2									
a_1	0.047	0.225	0.010	-	-	-	-0.989	0.398	0.060
a_2	-0.003	0.021	0.000	-	-	-	0.461	0.036	0.000
p_c	-0.006	0.003	0.000	-0.005	0.004	0.000	-	-	-
p_h	-0.000	0.008	0.000	0.003	0.009	0.000	-	-	-
K = 5									
a_1	0.032	0.191	0.010	-	-	-	-1.346	0.338	0.000
a_2	-0.002	0.018	0.000	-	-	-	0.492	0.032	0.000
p_c	-0.005	0.003	0.000	-0.004	0.004	0.000	-	-	-
p_h	-0.000	0.007	0.000	0.002	0.008	0.000	-	-	-
K = 10									
a_1	0.022	0.163	0.000	-	-	-	-1.640	0.286	0.000
a_2	-0.001	0.017	0.000	-	-	-	0.522	0.029	0.000
p_c	-0.005	0.003	0.000	-0.004	0.004	0.000	-	-	-
p_h	-0.001	0.007	0.000	0.001	0.007	0.000	-	-	-
M = 5	MB	R	Z	MB	R	Z	MB	R	Z
a_1	0.033	0.233	0.020	-	-	-	-0.730	0.421	0.150
a_2	-0.003	0.021	0.000	-	-	-	0.440	0.037	0.000
p_c	-0.007	0.003	0.000	-0.006	0.004	0.000	-	-	-
p_h	-0.002	0.008	0.000	0.000	0.008	0.000	-	-	-
K = 2									
a_1	0.044	0.209	0.000	-	-	-	-1.168	0.368	0.000
a_2	-0.003	0.020	0.000	-	-	-	0.474	0.034	0.000
p_c	-0.005	0.004	0.000	-0.005	0.004	0.000	-	-	-
p_h	-0.001	0.008	0.000	0.002	0.008	0.000	-	-	-
K = 5									
a_1	0.031	0.159	0.000	-	-	-	-1.642	0.283	0.000
a_2	-0.002	0.017	0.000	-	-	-	0.519	0.029	0.000
p_c	-0.004	0.003	0.000	-0.004	0.004	0.000	-	-	-
p_h	-0.001	0.006	0.000	0.001	0.007	0.000	-	-	-
K = 10									
a_1	0.020	0.125	0.000	-	-	-	-2.058	0.237	0.000
a_2	-0.001	0.014	0.000	-	-	-	0.572	0.026	0.000

Continued on next page

	Model 1			Model 2			Model 3		
p_c	-0.003	0.003	0.000	-0.003	0.003	0.000	-	-	-
p_h	-0.002	0.005	0.000	-0.001	0.005	0.000	-	-	-
M = 10	MB	R	Z	MB	R	Z	MB	R	Z
K = 1									
a_1	0.028	0.234	0.020	-	-	-	-0.757	0.428	0.170
a_2	-0.002	0.021	0.000	-	-	-	0.445	0.038	0.000
p_c	-0.006	0.003	0.000	-0.006	0.003	0.000	-	-	-
p_h	-0.004	0.007	0.000	-0.003	0.007	0.000	-	-	-
K = 2									
a_1	0.060	0.188	0.000	-	-	-	-1.466	0.333	0.000
a_2	-0.004	0.018	0.000	-	-	-	0.500	0.032	0.000
p_c	-0.005	0.003	0.000	-0.004	0.004	0.000	-	-	-
p_h	-0.002	0.007	0.000	0.001	0.007	0.000	-	-	-
K = 5									
a_1	0.028	0.131	0.000	-	-	-	-1.954	0.242	0.000
a_2	-0.002	0.015	0.000	-	-	-	0.557	0.026	0.000
p_c	-0.004	0.003	0.000	-0.003	0.003	0.000	-	-	-
p_h	-0.001	0.005	0.000	-0.000	0.005	0.000	-	-	-
K = 10									
a_1	0.022	0.101	0.000	-	-	-	-2.290	0.241	0.000
a_2	-0.001	0.012	0.000	-	-	-	0.612	0.027	0.000
p_c	-0.003	0.002	0.000	-0.002	0.002	0.000	-	-	-
p_h	-0.002	0.004	0.000	-0.001	0.004	0.000	-	-	-

S2 Case Studies

S2.1 Zebra mussels: single time point

S2.1.1 Site names

Table S6 shows the site name corresponding to the site code used in Figure 5.

Table S6: Site codes and corresponding site names.

Site code	Site
L1	Eight Acre Lake
L2	Farnham Lake
L3	Holme Pierrepont
L4	Pugneys Country Park
L5	Willen Lake
C1	Gloucester and Sharpness Canal
C2	Grand Union Canal
C3	Leeds and Liverpool Canal
C4	Shropshire Union Canal
C5	Worcester and Birmingham Canal
R1	Chasewater Reservoir
R2	Rutland Water Reservoir
R3	Staunton Harold Reservoir
R4	Ulley Reservoir
R5	Walthamstow Reservoir
Ri1	Little Ouse River
Ri2	River Nene
Ri3	Thames River
Ri4	River Weaver
Ri5	River Severn

S2.1.2 Model implementation

Table S7 shows the prior distributions used for this case study. For more details of the prior distribution for $\beta_{b,0}$ see Section S4.2.

The MCMC was run for 3 chains, each of 110,000 iterations, with a burn-in of 10,000, and thinning of 10. Table S8 shows the Gelman-Rubin statistic and the effective sample size (ESS) (gelman.diag() and effectiveSize() respectively in R [6]) for each parameter.

Table S7: Table of prior distributions used in the simulations of Section 4.1. Columns indicate the parameter name, its use in the model, and the prior distribution used. Inverse Gamma distribution uses the shape, scale parametrisation.

Parameter	Use	Prior
$\boldsymbol{\beta}_b$	Coefficients on site covariates	$\sim \mathrm{N}(0, 1)$
$\boldsymbol{\beta}_w$	Coefficients on sample covariates	$\sim \mathrm{N}(0, 1)$
$\boldsymbol{\beta}_{b,0}$	Intercept on log-DNA at $t = 1$	$\sim$ Exp-Unif$(0, \exp(20))$
τ^2	Variance of log-DNA across time	$\sim$ InvGamma$(2, 1)$
τ_1^2	Variance of log-DNA across sites	$\sim$ InvGamma$(2, 1)$
σ^2	Variance of log-DNA across samples	$\sim$ InvGamma$(2, 1)$
$(p_c, 1 - p_c - p_h, p_h)$	Probability of contamination or inhibition	$\sim$ Dirichlet$(0.01, 0.98, 0.01)$
a_1	CT log-variance intercept term	$\sim \mathrm{N}(0, 10^2)$
a_2	CT log-variance slope term	$\sim \mathrm{N}(0, 10^2)$
α_p^1	Plate regression intercepts	$\sim \mathrm{N}(0, 100^2)$
α_p^2	Plate regression slopes	$\sim \mathrm{N}(0, 100^2)$
σ_c	Standard deviation for contaminated or inhibited replicate	30
CT.max	Maximum CT value	40

Table S8: The Gelman-Rubin statistic (GR) and effective sample size (ESS) for simulation parameters for case study of Section 4.1.

Parameter	GR	ESS
a_1	1.00	2687.21
a_2	1.00	3176.11
α_1^1	1.02	434.24
α_2^1	1.02	243.79
α_3^1	1.01	356.77
α_4^1	1.01	465.05
α_5^1	1.01	302.14
α_6^1	1.01	536.91
α_7^1	1.00	328.48
α_8^1	1.01	513.63
α_9^1	1.01	415.70
α_{10}^1	1.00	700.40
α_{11}^1	1.00	334.94
α_{12}^1	1.00	699.97
α_{13}^1	1.01	499.97
α_{14}^1	1.01	873.01
α_{15}^1	1.00	661.22
α_{16}^1	1.00	518.45
α_{17}^1	1.02	423.62
α_{18}^1	1.01	328.71
α_{19}^1	1.00	674.42

Continued on next page

Parameter	GR	ESS
α_{20}^{1}	1.00	609.00
α_{1}^{2}	1.02	425.15
α_{2}^{2}	1.02	247.64
α_{3}^{2}	1.01	368.32
α_{4}^{2}	1.01	466.15
α_{5}^{2}	1.00	299.94
α_{6}^{2}	1.01	529.99
α_{7}^{2}	1.00	333.80
α_{8}^{2}	1.01	505.43
α_{9}^{2}	1.01	424.12
α_{10}^{2}	1.00	747.27
α_{11}^{2}	1.00	331.27
α_{12}^{2}	1.00	705.10
α_{13}^{2}	1.01	520.35
α_{14}^{2}	1.01	885.48
α_{15}^{2}	1.00	651.93
α_{16}^{2}	1.00	528.18
α_{17}^{2}	1.02	422.48
α_{18}^{2}	1.01	337.25
α_{19}^{2}	1.00	670.87
α_{20}^{2}	1.00	612.73
$\boldsymbol{\beta}_b[1]$	1.00	27391.14
$\boldsymbol{\beta}_b[2]$	1.00	25810.20
$\boldsymbol{\beta}_b[3]$	1.00	27362.67
$\beta_{b,0}$	1.00	15195.97
$\boldsymbol{\beta}_w[1]$	1.00	28881.66
$\boldsymbol{\beta}_w[2]$	1.00	16089.99
$\boldsymbol{\beta}_w[3]$	1.00	8332.48
$\boldsymbol{\beta}_w[4]$	1.00	26609.92
$\boldsymbol{\beta}_w[5]$	1.00	10617.71
$\boldsymbol{\beta}_w[6]$	1.00	15730.21
$\boldsymbol{\beta}_w[7]$	1.00	17248.86
l_1	1.00	11875.06
l_2	1.00	20138.16
l_3	1.00	17150.53
l_4	1.00	15689.56
l_5	1.00	10998.89
l_6	1.00	11092.51
l_7	1.00	13893.97
l_8	1.00	14232.93
l_9	1.00	13335.28
l_{10}	1.00	15924.76
l_{11}	1.00	16160.16

Continued on next page

Parameter	GR	ESS
l_{12}	1.00	10498.75
l_{13}	1.00	13391.14
l_{14}	1.00	15653.30
l_{15}	1.00	9581.43
l_{16}	1.00	15073.71
l_{17}	1.00	15357.04
l_{18}	1.00	14522.18
l_{19}	1.00	14728.96
l_{20}	1.00	13836.85
p_c	1.00	13433.83
p_h	1.00	3777.60
σ	1.00	28343.08
τ	1.00	26125.84

S2.2 Zebra mussels: multiple time points

S2.2.1 CT plot

Figure S1 shows the relationship between the standards' CT values and log-DNA for this data set. We can see that the CT values and log-DNA have a negative linear relationship, with small variation per plate in the fit (linear modelling using dglm() [8]). We can also see that the residuals of the fit increase as the log-DNA in the sample decreases.

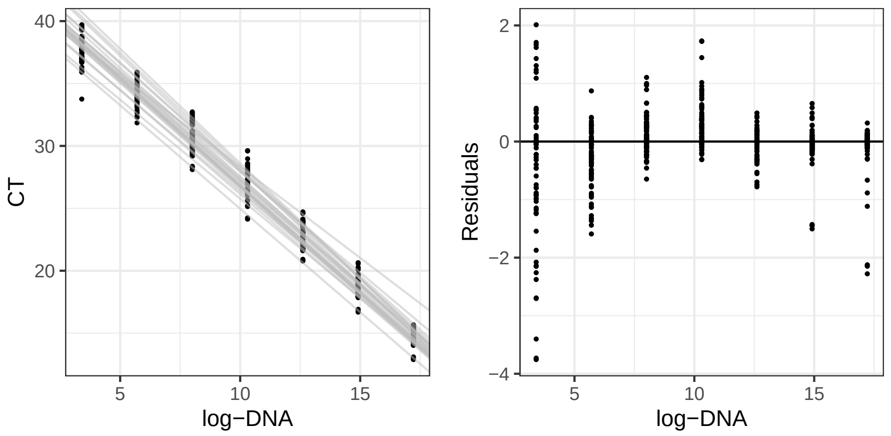

(a) (b)

Fig. S1: (a) CT values from standards against log-DNA in the standard. Each line is the linear fit of CT against log-DNA for each of the 24 plates used to analyse the samples. (b) Residuals of the linear regression with CT as the response and log-DNA concentration as the covariate

S2.2.2 Model implementation

Table S9 shows the prior distributions used for this case study. For more details of the prior distribution for $\beta_{b,0}$ see Section S4.2.

The MCMC was run for 3 chains, each of 110,000 iterations, with a burn-in of 10,000, and thinning of 10. Table S10 shows the Gelman-Rubin statistic and the effective sample size (ESS) (gelman.diag() and effectiveSize() respectively in R [6]) for each parameter.

Table S9: Table of prior distributions used in the simulations of Section 4.2. Columns indicate the parameter name, its use in the model, and the prior distribution used. Inverse Gamma distribution uses the shape, scale parametrisation.

Parameter	Use	Prior
$\boldsymbol{\beta}_b$	Coefficients on site covariates	$\sim$ N$(0, 1)$
$\boldsymbol{\beta}_w$	Coefficients on sample covariates	$\sim$ N$(0, 1)$
$\boldsymbol{\beta}_{b,0}$	Intercept on log-DNA at $t = 1$	$\sim$ Exp-Unif$(0, \exp(20))$
τ^2	Variance of log-DNA across time	$\sim$ InvGamma$(2, 1)$
τ_1^2	Variance of log-DNA across sites	$\sim$ InvGamma$(2, 1)$
σ^2	Variance of log-DNA across samples	$\sim$ InvGamma$(2, 1)$
$(p_c, 1 - p_c - p_h, p_h)$	Probabilities for contamination or inhibition	$\sim$ Dirichlet$(0.01, 0.98, 0.01)$
a_1	CT log-variance intercept term	$\sim$ N$(0, 10^2)$
a_2	CT log-variance slope term	$\sim$ N$(0, 10^2)$
α_p^1	Plate regression intercepts	$\sim$ N$(0, 100^2)$
α_p^2	Plate regression slopes	$\sim$ N$(0, 100^2)$
ρ_0	AR(1) coefficient mean	$\sim$ N$(1, 1)$
σ_ρ^2	AR(1) coefficient noise	$\sim$ InvGamma$(2, .1)$
σ_c	Standard deviation for contaminated or inhibited replicates	30
CT.max	Maximum CT value	40

Table S10: The Gelman-Rubin statistic (GR) and effective sample size (ESS) for simulation parameters for case study of Section 4.2

Parameter	GR	ESS
a_1	1.02	1325.32
a_2	1.01	1840.45
α_1^1	1.00	719.32
α_2^1	1.03	375.94
α_3^1	1.00	718.99
α_4^1	1.01	668.93
α_5^1	1.01	632.43
α_6^1	1.00	409.50
α_7^1	1.01	163.57
α_8^1	1.01	640.61
α_9^1	1.00	645.85
α_{10}^1	1.01	632.83
α_{11}^1	1.00	652.13
α_{12}^1	1.03	327.57
α_{13}^1	1.01	700.69
α_{14}^1	1.01	326.91
α_{15}^1	1.00	627.98
α_{16}^1	1.01	749.79
α_{17}^1	1.00	734.25
α_{18}^1	1.00	388.83
α_{19}^1	1.00	681.51

Continued on next page

Parameter	GR	ESS
α^1_{20}	1.00	828.48
α^1_{21}	1.00	677.33
α^1_{22}	1.00	614.94
α^1_{23}	1.01	585.32
α^1_{24}	1.01	609.85
α^2_{1}	1.00	712.77
α^2_{2}	1.03	384.41
α^2_{3}	1.00	718.83
α^2_{4}	1.01	672.33
α^2_{5}	1.01	602.81
α^2_{6}	1.00	420.56
α^2_{7}	1.01	166.55
α^2_{8}	1.01	646.84
α^2_{9}	1.00	643.63
α^2_{10}	1.02	648.22
α^2_{11}	1.00	661.29
α^2_{12}	1.02	326.31
α^2_{13}	1.01	693.74
α^2_{14}	1.01	325.55
α^2_{15}	1.00	632.83
α^2_{16}	1.01	747.87
α^2_{17}	1.00	745.76
α^2_{18}	1.00	397.82
α^2_{19}	1.00	681.91
α^2_{20}	1.00	832.32
α^2_{21}	1.00	672.82
α^2_{22}	1.00	595.94
α^2_{23}	1.01	587.38
α^2_{24}	1.01	640.53
$\beta_{b,0}$	1.00	24370.25
$\beta_w[1]$	1.00	15085.88
$\beta_w[2]$	1.00	26173.18
$\beta_w[3]$	1.00	9608.59
$l_{1,1}$	1.00	17276.89
$l_{2,1}$	1.00	6217.58
$l_{1,2}$	1.00	16633.03
$l_{2,2}$	1.00	1921.14
$l_{1,3}$	1.00	15334.31
$l_{2,3}$	1.00	4863.17
$l_{1,4}$	1.00	12680.03
$l_{2,4}$	1.00	5338.54
$l_{1,5}$	1.00	12019.59
$l_{2,5}$	1.00	4033.28

Continued on next page

Parameter	GR	ESS
$l_{1,6}$	1.00	14326.73
$l_{2,6}$	1.00	11171.64
$l_{1,7}$	1.00	9139.86
$l_{2,7}$	1.00	12339.21
$l_{1,8}$	1.00	19728.30
$l_{2,8}$	1.00	14820.63
$l_{1,9}$	1.00	13872.69
$l_{2,9}$	1.00	17038.97
$l_{1,10}$	1.00	17754.48
$l_{2,10}$	1.00	16884.42
$l_{1,11}$	1.00	16563.80
$l_{2,11}$	1.00	15610.48
$l_{1,12}$	1.00	17522.79
$l_{2,12}$	1.00	12778.40
p_c	1.00	21986.71
p_h	1.02	801.43
ρ_1	1.00	29383.59
ρ_2	1.00	24417.26
σ	1.00	16821.28
τ	1.00	15321.00
τ_1	1.00	27372.01

S2.3 Great crested newts

S2.3.1 CT plot

Figure S2 shows the relationship between the standards' CT values and log-DNA for this data set. We can see that the CT values and log-DNA have a negative linear relationship, with small variation per plate in the fit (linear modelling using dglm() [8]). There is a much smaller range of standards in this data set, and the variability in the fits per plate is much larger than in the other two data sets. The increase in residuals is also not as evident because the range of standards is not large enough to see this effect.

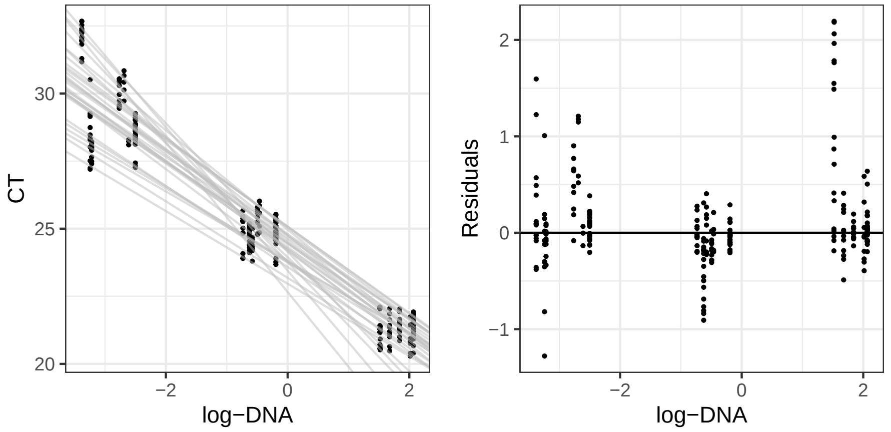

(a) (b)

Fig. S2: Great crested newts: (a) CT values from standards against log-DNA in the standard. Each line is the linear fit of CT against log-DNA for each of the 28 plates used to analyse the samples. (b) Residuals of the linear regression with CT as the response and log-DNA concentration as the covariate

S2.3.2 Model implementation

Table S11 shows the prior distributions used for this case study. For more details of the prior distribution for $\beta_{b,0}$ see Section S4.2.

For the other two case studies (for zebra mussels) CT.max was set at 40 during PCR analysis. For this case study (for great crested newts) it was set to 55. This was following protocol set by Biggs et al. [1]. However, upon inspection of the CT values from environmental samples, 95% of CT values lie in the interval $(33.020, 40.688)$, and only 93 of 1792 replicates amplified in the interval $(40, 55)$. If a sample did not amplify by cycle 40, then it was highly unlikely to amplify at all. It has been suggested that this limit is likely to be the point by which a single copy of DNA is expected to have been amplified enough to show sufficient fluorescence for detection. As there exists,

essentially, an upper limit to CT values (limited by the fact that the minimum number of DNA copies in a replicate that will amplify is 1), and to remain consistent with the other case studies, we set CT.max to 40, and set any CT values above this to NA.

Table S11: Table of prior distributions used in the simulations of Section 4.3. Columns indicate the parameter name, its use in the model, and the prior distribution used. Inverse Gamma distribution uses the shape, scale parametrisation.

Parameter	Use	Prior
β_b	Coefficients on site covariates	$\sim$ N(0, 1)
β_w	Coefficients on sample covariates	$\sim$ N(0, 1)
$\beta_{b,0}$	Intercept on log-DNA at $t = 1$	$\sim$ Exp-Unif(0, exp(20))
τ^2	Variance of log-DNA across time	$\sim$ InvGamma(2, 1)
τ_1^2	Variance of log-DNA across sites	$\sim$ InvGamma(2, 1)
σ^2	Variance of log-DNA across samples	$\sim$ InvGamma(2, 1)
$(p_c, 1 - p_c - p_h, p_h)$	Probability of contamination or inhibition	$\sim$ Dirichlet(0.01, 0.98, 0.01)
a_1	CT log-variance intercept term	$\sim$ N(0, 10^2)
a_2	CT log-variance slope term	$\sim$ N(0, 10^2)
α_p^1	Plate regression intercepts	$\sim$ N(0, 100^2)
α_p^2	Plate regression slopes	$\sim$ N(0, 100^2)
ρ_0	AR(1) coefficient mean	$\sim$ N(1, 1)
σ_ρ^2	AR(1) coefficient noise	$\sim$ InvGamma(2, .1)
σ_c	Standard deviation for contaminated or inhibited replicate	30
CT.max	Maximum CT value	40

The MCMC was run for 3 chains, each of 110,000 iterations, with a burn-in of 10,000, and thinning of 10. Table S12 shows the Gelman-Rubin statistic and the effective sample size (ESS) (gelman.diag() and effectiveSize() respectively in R [6]) for each parameter.

Table S12: The Gelman-Rubin statistic (GR) and effective sample size (ESS) for simulation parameters for case study of Section 4.3

Parameter	GR	ESS
a_1	1.00	1807.23
a_2	1.00	2516.23
α_1^1	1.00	9202.04
α_2^1	1.00	9826.67
α_3^1	1.00	7069.35
α_4^1	1.00	9381.11
α_5^1	1.00	9440.83
α_6^1	1.00	9478.53
α_7^1	1.00	13599.57
α_8^1	1.00	22192.37
α_9^1	1.00	23578.40

Continued on next page

Parameter	GR	ESS
α_{10}^{1}	1.00	15664.87
α_{11}^{1}	1.00	17127.00
α_{12}^{1}	1.00	3552.50
α_{13}^{1}	1.00	693.87
α_{14}^{1}	1.00	939.84
α_{15}^{1}	1.00	513.34
α_{16}^{1}	1.00	2253.25
α_{17}^{1}	1.00	9379.92
α_{18}^{1}	1.00	8126.83
α_{19}^{1}	1.00	8513.70
α_{20}^{1}	1.00	6561.17
α_{21}^{1}	1.00	9259.59
α_{22}^{1}	1.00	16058.55
α_{23}^{1}	1.00	16825.89
α_{24}^{1}	1.00	16458.24
α_{25}^{1}	1.00	17418.60
α_{26}^{1}	1.00	11203.40
α_{27}^{1}	1.00	10520.42
α_{28}^{1}	1.00	8677.66
α_{1}^{2}	1.00	2959.23
α_{2}^{2}	1.00	3035.22
α_{3}^{2}	1.00	2785.82
α_{4}^{2}	1.00	3029.58
α_{5}^{2}	1.00	3287.62
α_{6}^{2}	1.00	2947.93
α_{7}^{2}	1.00	1937.50
α_{8}^{2}	1.00	3908.41
α_{9}^{2}	1.00	5742.52
α_{10}^{2}	1.00	3378.12
α_{11}^{2}	1.00	2556.05
α_{12}^{2}	1.01	1165.69
α_{13}^{2}	1.00	530.13
α_{14}^{2}	1.01	785.93
α_{15}^{2}	1.00	370.55
α_{16}^{2}	1.00	826.38
α_{17}^{2}	1.00	2504.36
α_{18}^{2}	1.00	2281.66
α_{19}^{2}	1.00	2463.19
α_{20}^{2}	1.01	2213.79
α_{21}^{2}	1.00	2547.21
α_{22}^{2}	1.00	3000.92
α_{23}^{2}	1.00	4716.05
α_{24}^{2}	1.00	5067.63

Continued on next page

Parameter	GR	ESS
α^2_{25}	1.00	4882.36
α^2_{26}	1.00	3763.54
α^2_{27}	1.00	3445.20
α^2_{28}	1.00	3143.45
$\boldsymbol{\beta}_b[1]$	1.00	21253.85
$\boldsymbol{\beta}_b[2]$	1.00	14947.65
$\beta_{b,0}$	1.00	16977.03
$\boldsymbol{\beta}_w[1]$	1.00	9595.41
$\boldsymbol{\beta}_w[2]$	1.00	8849.71
$\boldsymbol{\beta}_w[3]$	1.00	19845.11
$\boldsymbol{\beta}_w[4]$	1.00	19862.28
$\boldsymbol{\beta}_w[5]$	1.00	14609.10
$\boldsymbol{\beta}_w[6]$	1.00	14374.89
$\boldsymbol{\beta}_w[7]$	1.00	17758.76
$\boldsymbol{\beta}_w[8]$	1.00	12291.17
$\boldsymbol{\beta}_w[9]$	1.00	14388.16
$l_{1,1}$	1.00	8684.84
$l_{1,2}$	1.00	8972.31
$l_{1,3}$	1.00	8368.51
$l_{1,4}$	1.00	8212.09
$l_{1,5}$	1.00	8395.19
$l_{1,6}$	1.00	6054.22
$l_{1,7}$	1.00	8237.65
$l_{1,8}$	1.00	2121.94
$l_{1,9}$	1.00	2160.92
$l_{1,10}$	1.00	3161.21
$l_{1,11}$	1.00	9446.42
$l_{1,12}$	1.00	9830.04
$l_{1,13}$	1.00	10964.65
$l_{1,14}$	1.00	9042.48
$l_{1,15}$	1.00	8958.75
$l_{1,16}$	1.00	7544.21
$l_{1,17}$	1.00	6793.86
$l_{1,18}$	1.00	4784.50
$l_{1,19}$	1.00	5761.66
p_c	1.00	27711.05
p_h	1.00	392.49
ρ	1.00	28710.56
σ	1.00	7491.15
τ	1.00	8473.63
τ_1	1.00	3416.10

S3 Comparison to existing methods

Existing methods for analysing qPCR data where CT values are linked to log-DNA concentrations whilst accounting for the truncation in observations at CT.max include [4] and [9]. Both model the log-DNA concentration as a function of covariates. The latter includes several modelling stages, from DNA availability, DNA collection, and DNA analysis. The former models log-DNA concentration directly from CT values, where the plate regression coefficients α_p^1, α_p^2 are assumed to be known and fixed quantities (where these are taken from the qPCR analysis output). Neither model accounts for the variance heteroscedasticity of CT values across log-DNA concentrations in the replicates, nor do they account for possible contamination and inhibition of replicates. We showed in Section 3.1 that ignoring these when they are present can lead to increased bias in model output.

Analysis can be conducted using the back-transformed CT values directly [2]. However, this analysis often involves several key assumptions. Firstly, that missing observations are random. In reality, missing observations are not random and are in fact indicative of the concentration in the replicate. This can positively bias log-DNA concentrations, as the replicates that fail to amplify do so precisely because they contain very little DNA. New modelling approaches resolve this by directly accounting for this truncation in the observation process.

Secondly, it does not account for uncertainty in the plate regression coefficients. These are assumed to be known and fixed for each plate based on a log-linear regression of the CT values for the standards. Therefore transformations of CT values do not fully account for uncertainty in the resulting log-DNA in the replicate. Furthermore, back-transforming CT values gives an estimate of the log-DNA in each replicate only. Combining estimates from all replicates into an estimation of log-DNA at a site is a non-trivial task. Often, CT values are averaged over replicates in a sample, or indeed samples over a site. Otherwise, a box plot of the expected back-transformed CT values of each replicate from a site may be produced. However, that these correctly account for the full uncertainty surrounding estimates of log-DNA and covariate coefficients is unclear.

S3.1 Comparison to analysis of back-transformed CT values

We include here an example analysis using the back-transformed CT values on simulated data to compare our method to a simpler analysis.

Our simulated data is as in Section 3. For the back-transformed CT values, we determine the plate parameters that would ordinarily be outputted by the lab by conducting a log-linear regression of the standards' CT values. To analyse the data, we use a linear mixed effects model with the back-transformed CT values as the response variable. The sample and site level covariates are included as fixed effects, site as a random effect, and time as a nested random effect. In the case of $(M, K) = (1, 1)$ time was included as a simple random effect to avoid problems of over-parametrising the model (these results are indicated by a $\star$ in the results tables). Models were fit in R [7] using the lmer function [3], and model predictions were obtained using the predictInterval function from [5]. Predictions for log-DNA concentrations were made

with sample level covariates set to zero or the reference level in order to be consistent with Model 1.

Table S13 shows results for MSE, mean width of 95% confidence intervals, and corresponding mean coverage for $l_{i,t}$ in the cases $(p_c, p_h) = (0.05, 0.1)$ and $(p_c, p_h) = (0.01, 0.02)$. Tables S14 and S15 show results for MB, mean width of 95% confidence intervals, and mean proportion of confidence intervals containing zero for the site and sampling coefficients $\boldsymbol{\beta}_b$ and $\boldsymbol{\beta}_w$ for $(p_c, p_h) = (0.05, 0.1)$ and $(p_c, p_h) = (0.01, 0.02)$ respectively.

From Table S13 we can see that the MSE increases when the probability of contamination and inhibition increases. We note that, often, with increasing replication in the replicates that the MSE increases. This is likely because the increase in replication increases the probability that one or more of those replicates is contaminated or inhibited. Without accounting for this process (as observed with Model 3 in Section 3.1) this can lead to increased bias. Comparing the results from Table S13 to those from Table 1, we can see that there is a significant increase in the MSE of model estimates and much larger confidence intervals. From Tables S14 and S15 we can also see that this model has increased bias in covariate estimates when compared to posteriors in Tables S2 and S3.

Table S13: Mean square error (MSE), mean range of 95% confidence interval (R), and mean coverage of $l_{i,t}$ (C) for the linear mixed effects model. Probability of contamination and inhibition $(p_c, p_h) = (0.05, 0.1)$ and $(p_c, p_h) = (0.01, 0.02)$.

$(p_c, p_h) = (0.05, 0.1)$			
M = 1	MSE	R	C
K = 1*	7.187	8.789	0.906
K = 2	5.917	5.599	0.832
K = 5	7.066	5.385	0.823
K = 10	8.304	5.281	0.818
M = 2	MSE	R	C
K = 1	4.490	6.996	0.897
K = 2	5.523	6.279	0.882
K = 5	6.006	5.891	0.881
K = 10	7.698	5.869	0.877
M = 5	MSE	R	C
K = 1	4.709	6.722	0.899
K = 2	4.980	6.416	0.903
K = 5	5.572	6.266	0.908
K = 10	6.781	6.155	0.904
M = 10	MSE	R	C
K = 1	5.318	6.615	0.905
K = 2	4.913	6.394	0.921
K = 5	6.689	6.275	0.901
K = 10	7.631	6.226	0.894

$(p_c, p_h) = (0.01, 0.02)$			
M = 1	MSE	R	C
K = 1*	6.079	8.109	0.911
K = 2	4.140	4.342	0.821
K = 5	4.516	4.039	0.809
K = 10	4.396	3.978	0.817
M = 2	MSE	R	C
K = 1	3.463	6.025	0.905
K = 2	3.677	5.279	0.894
K = 5	4.089	4.881	0.893
K = 10	4.634	4.832	0.890
M = 5	MSE	R	C
K = 1	2.963	5.733	0.922
K = 2	3.527	5.448	0.911
K = 5	3.587	5.240	0.927
K = 10	4.507	5.147	0.913
M = 10	MSE	R	C
K = 1	2.851	5.615	0.928
K = 2	3.296	5.412	0.928
K = 5	3.824	5.255	0.928
K = 10	4.702	5.614	0.930

Table S14: Mean bias (MB), the mean width of the 95% confidence intervals (R), and the mean number of intervals containing zero (Z) of the covariate coefficients $\boldsymbol{\beta}_b, \boldsymbol{\beta}_w$ for the linear mixed effects model under different sampling designs. $\boldsymbol{\beta}_b = (1, -1)$, $\boldsymbol{\beta}_w = (1, -1)$, and the probability of contamination and inhibition $(p_c, p_h) = (0.05, 0.1)$.

	M=1			M=2		
	MB	R	Z	MB	R	Z
K = 1*						
$\boldsymbol{\beta}_b[1]$	-0.302	0.700	0.040	-0.253	0.586	0.000
$\boldsymbol{\beta}_b[2]$	0.318	1.377	0.450	0.291	1.159	0.330
$\boldsymbol{\beta}_w[1]$	-0.303	0.704	0.060	-0.248	0.402	0.000
$\boldsymbol{\beta}_w[2]$	0.301	1.378	0.490	0.195	0.787	0.010
K = 2						
$\boldsymbol{\beta}_b[1]$	-0.348	0.652	0.040	-0.313	0.569	0.000
$\boldsymbol{\beta}_b[2]$	0.283	1.281	0.430	0.345	1.110	0.290
$\boldsymbol{\beta}_w[1]$	-0.337	0.654	0.030	-0.213	0.279	0.000
$\boldsymbol{\beta}_w[2]$	0.277	1.278	0.370	0.228	0.548	0.020
K = 5						
$\boldsymbol{\beta}_b[1]$	-0.398	0.626	0.070	-0.391	0.549	0.050
$\boldsymbol{\beta}_b[2]$	0.433	1.231	0.550	0.362	1.081	0.380
$\boldsymbol{\beta}_w[1]$	-0.415	0.626	0.130	-0.198	0.177	0.000
$\boldsymbol{\beta}_w[2]$	0.398	1.230	0.530	0.206	0.344	0.000
K = 10						
$\boldsymbol{\beta}_b[1]$	-0.480	0.627	0.210	-0.448	0.551	0.100
$\boldsymbol{\beta}_b[2]$	0.433	1.231	0.480	0.400	1.090	0.400
$\boldsymbol{\beta}_w[1]$	-0.436	0.625	0.110	-0.192	0.129	0.000
$\boldsymbol{\beta}_w[2]$	0.414	1.234	0.530	0.180	0.253	0.000

	M=5			M=10		
	MB	R	Z	MB	R	Z
K = 1						
$\boldsymbol{\beta}_b[1]$	-0.317	0.520	0.010	-0.369	0.508	0.030
$\boldsymbol{\beta}_b[2]$	0.329	1.030	0.290	0.312	1.001	0.220
$\boldsymbol{\beta}_w[1]$	-0.213	0.229	0.000	-0.210	0.155	0.000
$\boldsymbol{\beta}_w[2]$	0.224	0.445	0.000	0.215	0.302	0.000
K = 2						
$\boldsymbol{\beta}_b[1]$	-0.369	0.515	0.020	-0.340	0.485	0.040
$\boldsymbol{\beta}_b[2]$	0.327	1.014	0.270	0.348	0.965	0.230
$\boldsymbol{\beta}_w[1]$	-0.206	0.158	0.000	-0.196	0.106	0.000
$\boldsymbol{\beta}_w[2]$	0.213	0.308	0.000	0.193	0.206	0.000
K = 5						
$\boldsymbol{\beta}_b[1]$	-0.412	0.510	0.040	-0.424	0.470	0.030
$\boldsymbol{\beta}_b[2]$	0.394	1.001	0.320	0.434	0.935	0.340
$\boldsymbol{\beta}_w[1]$	-0.202	0.100	0.000	-0.210	0.069	0.000
$\boldsymbol{\beta}_w[2]$	0.209	0.195	0.000	0.214	0.134	0.000
K = 10						

Continued on next page

	M=1			M=2		
$\boldsymbol{\beta}_b[1]$	-0.453	0.503	0.050	-0.509	0.481	0.100
$\boldsymbol{\beta}_b[2]$	0.501	0.983	0.510	0.515	0.958	0.510
$\boldsymbol{\beta}_w[1]$	-0.194	0.069	0.000	-0.223	0.049	0.000
$\boldsymbol{\beta}_w[2]$	0.211	0.136	0.000	0.213	0.095	0.000

Table S15: Mean bias (MB), the mean width of the 95% confidence intervals (R), and the mean number of intervals containing zero (Z) of the covariate coefficients $\boldsymbol{\beta}_b, \boldsymbol{\beta}_w$ for the linear mixed effects model under different sampling designs. $\boldsymbol{\beta}_b = (1, -1)$, $\boldsymbol{\beta}_w = (1, -1)$, and the probability of contamination and inhibition $(p_c, p_h) = (0.01, 0.02)$.

	M=1			M=2		
	MB	R	Z	MB	R	Z
K = 1*						
$\boldsymbol{\beta}_b[1]$	-0.233	0.631	0.000	-0.216	0.561	0.000
$\boldsymbol{\beta}_b[2]$	0.223	1.244	0.330	0.227	1.090	0.220
$\boldsymbol{\beta}_w[1]$	-0.211	0.640	0.010	-0.160	0.334	0.000
$\boldsymbol{\beta}_w[2]$	0.175	1.242	0.230	0.139	0.650	0.010
K = 2						
$\boldsymbol{\beta}_b[1]$	-0.227	0.636	0.000	-0.211	0.548	0.000
$\boldsymbol{\beta}_b[2]$	0.199	1.240	0.300	0.256	1.082	0.190
$\boldsymbol{\beta}_w[1]$	-0.211	0.646	0.020	-0.155	0.216	0.000
$\boldsymbol{\beta}_w[2]$	0.222	1.242	0.300	0.147	0.419	0.000
K = 5						
$\boldsymbol{\beta}_b[1]$	-0.266	0.592	0.000	-0.258	0.542	0.000
$\boldsymbol{\beta}_b[2]$	0.287	1.164	0.320	0.254	1.065	0.210
$\boldsymbol{\beta}_w[1]$	-0.237	0.604	0.000	-0.123	0.129	0.000
$\boldsymbol{\beta}_w[2]$	0.252	1.164	0.300	0.082	0.254	0.000
K = 10						
$\boldsymbol{\beta}_b[1]$	-0.272	0.590	0.040	-0.267	0.549	0.000
$\boldsymbol{\beta}_b[2]$	0.209	1.159	0.260	0.237	1.088	0.230
$\boldsymbol{\beta}_w[1]$	-0.273	0.592	0.010	-0.108	0.091	0.000
$\boldsymbol{\beta}_w[2]$	0.296	1.160	0.260	0.108	0.178	0.000
	M=5			M=10		
	MB	R	Z	MB	R	Z
K = 1						
$\boldsymbol{\beta}_b[1]$	-0.230	0.511	0.000	-0.226	0.495	0.010
$\boldsymbol{\beta}_b[2]$	0.212	1.012	0.160	0.224	0.980	0.160
$\boldsymbol{\beta}_w[1]$	-0.122	0.178	0.000	-0.121	0.120	0.000
$\boldsymbol{\beta}_w[2]$	0.121	0.346	0.000	0.122	0.233	0.000
K = 2						
$\boldsymbol{\beta}_b[1]$	-0.254	0.507	0.000	-0.260	0.480	0.000
$\boldsymbol{\beta}_b[2]$	0.287	0.991	0.190	0.283	0.945	0.190

Continued on next page

	M=1			M=2		
$\boldsymbol{\beta}_w[1]$	-0.121	0.123	0.000	-0.127	0.083	0.000
$\boldsymbol{\beta}_w[2]$	0.127	0.239	0.000	0.112	0.162	0.000
K = 5						
$\boldsymbol{\beta}_b[1]$	-0.253	0.511	0.010	-0.303	0.477	0.010
$\boldsymbol{\beta}_b[2]$	0.276	1.006	0.260	0.272	0.951	0.180
$\boldsymbol{\beta}_w[1]$	-0.103	0.074	0.000	-0.124	0.052	0.000
$\boldsymbol{\beta}_w[2]$	0.102	0.144	0.000	0.109	0.101	0.000
K = 10						
$\boldsymbol{\beta}_b[1]$	-0.321	0.504	0.000	-0.319	0.496	0.020
$\boldsymbol{\beta}_b[2]$	0.334	0.995	0.270	0.309	0.988	0.270
$\boldsymbol{\beta}_w[1]$	-0.123	0.053	0.000	-0.118	0.037	0.000
$\boldsymbol{\beta}_w[2]$	0.107	0.103	0.000	0.116	0.072	0.000

S4 Prior sensitivity

We use the case studies to analyse the sensitivity of the output to prior specification. In particular we look at the sensitivity of the model to the prior set on the plate regression coefficients α_p^1, α_p^2, a_1, a_2, and the intercept $\beta_{b,0}$ of $l_{i,1}$.

S4.1 Plate Regression Coefficients

We look at the posterior summaries for model parameters for the case studies from Sections 4.1 and 4.3 under several different prior specifications (the case study in Section 4.2 uses the same set of standards as 4.1).

Table S16 shows posterior means and 95% PCIs for the plate regression coefficients $\alpha_p^1, \alpha_p^2, a_1, a_2$ for the case study in Section 4.1. We consider four sets of prior specifications:

- Set 1: $\alpha_p^1 \sim \mathrm{N}(44.481, 1), \alpha_p^2 \sim \mathrm{N}(-1.715, 1), a_1 \sim \mathrm{N}(0.815, 1), a_2 \sim \mathrm{N}(-0.307, 1)$.
- Set 2: $\alpha_p^1 \sim \mathrm{N}(44, 5^2), \alpha_p^2 \sim \mathrm{N}(-1.7, 5^2), a_1 \sim \mathrm{N}(0.5, 1), a_2 \sim \mathrm{N}(0, 1)$.
- Set 3: $\alpha_p^1 \sim \mathrm{N}(44, 10^2), \alpha_p^2 \sim \mathrm{N}(-1.7, 10^2), a_1, a_2 \sim \mathrm{N}(0, 1)$.
- Set 4: $\alpha_p^1, \alpha_p^2 \sim \mathrm{N}(0, 100^2), a_1, a_2 \sim \mathrm{N}(0, 10^2)$.

Table S17 shows posterior means and 95% PCIs for the plate regression coefficients $\alpha_p^1, \alpha_p^2, a_1, a_2$ for the case study in Section 4.3. We again consider four sets of prior specifications:

- Set 1: $\alpha_p^1 \sim \mathrm{N}(24.178, 1), \alpha_p^2 \sim \mathrm{N}(-1.727, 1), a_1 \sim \mathrm{N}(-3.596, 1), a_2 \sim \mathrm{N}(-0.209, 1)$.
- Set 2: $\alpha_p^1 \sim \mathrm{N}(24, 5^2), \alpha_p^2 \sim \mathrm{N}(-1.7, 5^2), a_1 \sim \mathrm{N}(-3, 1), a_2 \sim \mathrm{N}(0, 1)$.
- Set 3: $\alpha_p^1 \sim \mathrm{N}(24, 10^2), \alpha_p^2 \sim \mathrm{N}(-1.7, 10^2), a_1, a_2 \sim \mathrm{N}(0, 1)$.
- Set 4: $\alpha_p^1, \alpha_p^2 \sim \mathrm{N}(0, 100^2), a_1, a_2 \sim \mathrm{N}(0, 10^2)$.

In both cases, the prior means in Set 1 are determined by a preliminary linear regression analysis of the data restricted to the standards. The other sets of priors consider increasing prior variances and deviations in the prior mean. Set 4 is the prior distribution used in previous analyses. Both Tables S16 and S17 show that the posterior distributions for the plate regression coefficients are not sensitive to these prior distributions.

Table S16: Posterior means and 95% PCIs for plate regression coefficients $\alpha_p^1, \alpha_p^2, a_1$, and a_2 for the case study from Section 4.1 under different prior specifications from Set 1 to Set4.

	Set 1	Set 2	Set 3	Set 4
α_1^1	43.422(43.139,43.742)	43.406(43.136,43.712)	43.409(43.115,43.729)	43.395(43.105,43.678)
α_2^1	45.526(45.245,45.8)	45.54(45.266,45.804)	45.526(45.261,45.794)	45.547(45.287,45.83)
α_3^1	43.08(42.817,43.333)	43.044(42.789,43.308)	43.056(42.801,43.322)	43.052(42.781,43.307)
α_4^1	42.024(41.775,42.272)	41.976(41.718,42.231)	41.995(41.759,42.245)	41.98(41.725,42.234)
α_5^1	46.37(46.056,46.687)	46.437(46.126,46.75)	46.438(46.157,46.742)	46.436(46.126,46.757)
α_6^1	44.102(43.773,44.425)	44.083(43.756,44.413)	44.08(43.744,44.395)	44.079(43.763,44.421)
α_7^1	45.985(45.692,46.332)	46.028(45.727,46.366)	46.025(45.749,46.337)	46.015(45.708,46.364)
α_8^1	43.773(43.505,44.039)	43.759(43.486,44.023)	43.749(43.469,44.015)	43.761(43.478,44.034)
α_9^1	44.021(43.654,44.347)	44.033(43.641,44.356)	43.992(43.565,44.337)	44.002(43.604,44.35)
α_{10}^1	44.961(44.697,45.222)	44.961(44.683,45.233)	44.969(44.711,45.235)	44.961(44.696,45.232)
α_{11}^1	43.748(43.493,44.018)	43.74(43.458,44.007)	43.715(43.438,43.99)	43.733(43.458,44.01)
α_{12}^1	44.938(44.656,45.207)	44.957(44.698,45.219)	44.945(44.678,45.214)	44.943(44.678,45.204)
α_{13}^1	45.541(45.265,45.821)	45.558(45.276,45.828)	45.573(45.288,45.848)	45.563(45.275,45.837)
α_{14}^1	44.863(44.612,45.121)	44.862(44.599,45.131)	44.87(44.598,45.126)	44.862(44.604,45.121)
α_{15}^1	43.942(43.702,44.192)	43.941(43.692,44.184)	43.945(43.676,44.196)	43.945(43.683,44.208)
α_{16}^1	44.728(44.453,45.028)	44.733(44.441,45.036)	44.727(44.45,45.032)	44.717(44.43,45.025)
α_{17}^1	44.282(44.032,44.545)	44.259(44.002,44.521)	44.25(43.982,44.512)	44.276(44.01,44.533)
α_{18}^1	45.533(45.262,45.805)	45.56(45.288,45.848)	45.541(45.284,45.812)	45.567(45.273,45.843)
α_{19}^1	43.302(43.046,43.551)	43.285(43.031,43.528)	43.275(43.016,43.529)	43.278(43.028,43.542)
α_{20}^1	45.561(45.305,45.815)	45.587(45.326,45.841)	45.592(45.33,45.868)	45.579(45.327,45.84)
α_1^2	-1.661(-1.685,-1.643)	-1.66(-1.682,-1.643)	-1.661(-1.684,-1.642)	-1.66(-1.678,-1.641)
α_2^2	-1.758(-1.775,-1.74)	-1.759(-1.775,-1.741)	-1.758(-1.775,-1.741)	-1.759(-1.777,-1.743)
α_3^2	-1.658(-1.674,-1.642)	-1.656(-1.672,-1.64)	-1.657(-1.673,-1.641)	-1.656(-1.672,-1.639)
α_4^2	-1.585(-1.601,-1.57)	-1.582(-1.598,-1.566)	-1.583(-1.599,-1.568)	-1.582(-1.598,-1.566)
α_5^2	-1.817(-1.837,-1.798)	-1.821(-1.841,-1.802)	-1.822(-1.84,-1.804)	-1.821(-1.841,-1.802)
α_6^2	-1.684(-1.704,-1.664)	-1.683(-1.703,-1.663)	-1.683(-1.702,-1.662)	-1.683(-1.703,-1.664)
α_7^2	-1.779(-1.801,-1.761)	-1.782(-1.803,-1.763)	-1.782(-1.801,-1.764)	-1.781(-1.803,-1.762)
α_8^2	-1.71(-1.726,-1.693)	-1.709(-1.725,-1.692)	-1.708(-1.725,-1.691)	-1.709(-1.726,-1.691)
α_9^2	-1.683(-1.703,-1.661)	-1.684(-1.704,-1.661)	-1.682(-1.703,-1.656)	-1.682(-1.703,-1.659)
α_{10}^2	-1.754(-1.77,-1.738)	-1.754(-1.771,-1.737)	-1.755(-1.771,-1.739)	-1.754(-1.771,-1.738)
α_{11}^2	-1.708(-1.725,-1.692)	-1.708(-1.724,-1.69)	-1.706(-1.723,-1.689)	-1.707(-1.725,-1.69)
α_{12}^2	-1.753(-1.769,-1.735)	-1.754(-1.77,-1.738)	-1.753(-1.77,-1.737)	-1.753(-1.769,-1.737)
α_{13}^2	-1.759(-1.777,-1.741)	-1.76(-1.777,-1.742)	-1.761(-1.778,-1.743)	-1.76(-1.778,-1.742)
α_{14}^2	-1.748(-1.764,-1.733)	-1.748(-1.765,-1.732)	-1.749(-1.765,-1.732)	-1.748(-1.764,-1.732)
α_{15}^2	-1.676(-1.691,-1.66)	-1.675(-1.691,-1.66)	-1.676(-1.692,-1.659)	-1.676(-1.692,-1.659)
α_{16}^2	-1.67(-1.689,-1.654)	-1.671(-1.689,-1.653)	-1.67(-1.689,-1.653)	-1.67(-1.689,-1.652)
α_{17}^2	-1.671(-1.688,-1.655)	-1.67(-1.686,-1.654)	-1.669(-1.686,-1.652)	-1.671(-1.687,-1.654)
α_{18}^2	-1.759(-1.776,-1.741)	-1.76(-1.778,-1.743)	-1.759(-1.776,-1.743)	-1.761(-1.778,-1.742)
α_{19}^2	-1.639(-1.655,-1.623)	-1.638(-1.654,-1.622)	-1.638(-1.654,-1.621)	-1.638(-1.654,-1.622)
α_{20}^2	-1.767(-1.783,-1.751)	-1.768(-1.784,-1.752)	-1.769(-1.786,-1.752)	-1.768(-1.784,-1.752)
a_1	0.616 (0.380,0.850)	0.623 (0.381, 0.854)	0.608 (0.362,0.849)	0.535 (0.305,0.758)
a_2	-0.404 (-0.426,-0.382)	-0.405 (-0.427, -0.383)	-0.404 (-0.427,-0.382)	-0.398 (-0.419,-0.376)

Table S17: Posterior means and 95% PCIs for plate regression coefficients $\alpha_p^1, \alpha_p^2, a_1$, and a_2 for the case study from Section 4.3 under different prior specifications from Set 1 to Set 4.

	Set 1	Set 2	Set 3	Set 4
α_1^1	24.679(24.552,24.811)	24.678(24.551,24.808)	24.677(24.544,24.809)	24.68(24.531,24.83)
α_2^1	24.776(24.65,24.904)	24.778(24.65,24.905)	24.775(24.644,24.906)	24.778(24.63,24.925)
α_3^1	24.525(24.398,24.652)	24.525(24.395,24.657)	24.522(24.391,24.653)	24.528(24.381,24.678)
α_4^1	24.643(24.512,24.774)	24.643(24.513,24.773)	24.644(24.514,24.776)	24.649(24.5,24.798)
α_5^1	24.29(24.164,24.418)	24.288(24.16,24.416)	24.287(24.159,24.418)	24.293(24.146,24.44)
α_6^1	24.796(24.671,24.924)	24.799(24.669,24.926)	24.797(24.667,24.926)	24.801(24.655,24.949)
α_7^1	24.26(24.077,24.413)	24.261(24.089,24.412)	24.264(24.099,24.415)	24.284(24.115,24.445)
α_8^1	23.747(23.619,23.875)	23.744(23.613,23.873)	23.741(23.609,23.871)	23.744(23.596,23.892)
α_9^1	22.994(22.862,23.123)	22.988(22.857,23.118)	22.986(22.852,23.117)	22.99(22.84,23.138)
α_{10}^1	24.061(23.934,24.189)	24.061(23.932,24.191)	24.059(23.927,24.19)	24.061(23.912,24.21)
α_{11}^1	23.822(23.696,23.949)	23.82(23.694,23.948)	23.819(23.688,23.948)	23.818(23.669,23.965)
α_{12}^1	23.855(23.703,24.01)	23.849(23.695,24.005)	23.84(23.684,23.996)	23.855(23.676,24.037)
α_{13}^1	23.432(23.281,23.586)	23.426(23.273,23.581)	23.419(23.262,23.575)	23.436(23.26,23.619)
α_{14}^1	23.107(22.947,23.269)	23.099(22.94,23.262)	23.093(22.931,23.255)	23.093(22.908,23.28)
α_{15}^1	23.793(23.621,23.98)	23.791(23.618,23.982)	23.779(23.605,23.96)	23.874(23.647,24.112)
α_{16}^1	23.375(23.073,23.684)	23.334(23.032,23.638)	24.124(23.96,24.289)	23.336(22.99,23.684)
α_{17}^1	24.536(24.407,24.665)	24.537(24.409,24.665)	24.531(24.398,24.661)	24.545(24.396,24.695)
α_{18}^1	25.216(25.086,25.346)	25.219(25.089,25.349)	25.219(25.086,25.353)	25.229(25.079,25.377)
α_{19}^1	24.983(24.854,25.112)	24.987(24.859,25.116)	24.986(24.855,25.115)	24.992(24.847,25.142)
α_{20}^1	24.318(24.174,24.453)	24.32(24.178,24.455)	24.319(24.175,24.457)	24.328(24.177,24.479)
α_{21}^1	24.928(24.798,25.058)	24.934(24.804,25.066)	24.933(24.802,25.064)	24.944(24.796,25.091)
α_{22}^1	23.676(23.538,23.813)	23.673(23.535,23.812)	23.67(23.53,23.81)	23.675(23.512,23.844)
α_{23}^1	23.811(23.667,23.945)	23.809(23.663,23.941)	23.809(23.666,23.944)	23.815(23.665,23.963)
α_{24}^1	23.197(23.07,23.324)	23.191(23.064,23.319)	23.188(23.058,23.317)	23.188(23.039,23.336)
α_{25}^1	23.602(23.451,23.749)	23.599(23.445,23.744)	23.605(23.45,23.749)	23.624(23.469,23.774)
α_{26}^1	24.435(24.307,24.563)	24.435(24.307,24.562)	24.434(24.304,24.565)	24.427(24.281,24.574)
α_{27}^1	25.062(24.931,25.192)	25.065(24.935,25.196)	25.063(24.932,25.195)	25.063(24.913,25.214)
α_{28}^1	25.054(24.923,25.184)	25.057(24.927,25.188)	25.055(24.923,25.189)	25.053(24.905,25.206)
α_1^2	-1.525(-1.593,-1.459)	-1.524(-1.591,-1.457)	-1.522(-1.592,-1.453)	-1.526(-1.604,-1.451)
α_2^2	-1.539(-1.605,-1.473)	-1.539(-1.605,-1.472)	-1.536(-1.605,-1.468)	-1.537(-1.611,-1.462)
α_3^2	-1.529(-1.597,-1.462)	-1.528(-1.598,-1.46)	-1.526(-1.595,-1.457)	-1.533(-1.612,-1.456)
α_4^2	-1.574(-1.643,-1.506)	-1.573(-1.643,-1.505)	-1.574(-1.645,-1.505)	-1.578(-1.656,-1.502)
α_5^2	-1.603(-1.67,-1.539)	-1.601(-1.667,-1.536)	-1.601(-1.667,-1.535)	-1.609(-1.682,-1.536)
α_6^2	-1.585(-1.651,-1.521)	-1.585(-1.652,-1.519)	-1.585(-1.652,-1.518)	-1.587(-1.663,-1.513)
α_7^2	-1.555(-1.653,-1.467)	-1.551(-1.644,-1.461)	-1.547(-1.634,-1.46)	-1.557(-1.644,-1.466)
α_8^2	-1.437(-1.501,-1.375)	-1.435(-1.5,-1.372)	-1.429(-1.493,-1.367)	-1.432(-1.504,-1.362)
α_9^2	-1.38(-1.445,-1.316)	-1.379(-1.444,-1.315)	-1.376(-1.441,-1.312)	-1.382(-1.455,-1.312)
α_{10}^2	-1.568(-1.634,-1.503)	-1.568(-1.635,-1.503)	-1.565(-1.635,-1.497)	-1.568(-1.644,-1.493)
α_{11}^2	-1.68(-1.757,-1.604)	-1.68(-1.758,-1.604)	-1.677(-1.755,-1.6)	-1.675(-1.761,-1.59)
α_{12}^2	-1.716(-1.821,-1.618)	-1.712(-1.816,-1.61)	-1.704(-1.804,-1.602)	-1.718(-1.836,-1.605)
α_{13}^2	-1.678(-1.78,-1.585)	-1.675(-1.778,-1.578)	-1.665(-1.768,-1.566)	-1.683(-1.806,-1.574)
α_{14}^2	-1.62(-1.735,-1.512)	-1.617(-1.73,-1.509)	-1.611(-1.723,-1.5)	-1.609(-1.737,-1.485)
α_{15}^2	-2.016(-2.191,-1.896)	-2.013(-2.181,-1.89)	-1.995(-2.148,-1.872)	-2.088(-2.278,-1.911)
α_{16}^2	-2.559(-2.727,-2.386)	-2.583(-2.742,-2.418)	-1.539(-1.654,-1.429)	-2.584(-2.769,-2.405)
α_{17}^2	-1.771(-1.842,-1.7)	-1.77(-1.84,-1.7)	-1.765(-1.837,-1.692)	-1.78(-1.863,-1.698)
α_{18}^2	-1.795(-1.867,-1.722)	-1.795(-1.867,-1.723)	-1.797(-1.872,-1.724)	-1.807(-1.889,-1.726)
α_{19}^2	-1.845(-1.919,-1.774)	-1.846(-1.918,-1.774)	-1.845(-1.919,-1.773)	-1.849(-1.931,-1.767)
α_{20}^2	-1.84(-1.917,-1.754)	-1.841(-1.917,-1.757)	-1.841(-1.92,-1.755)	-1.85(-1.935,-1.762)
α_{21}^2	-1.947(-2.019,-1.872)	-1.95(-2.023,-1.876)	-1.95(-2.023,-1.876)	-1.961(-2.042,-1.882)
α_{22}^2	-1.434(-1.502,-1.366)	-1.432(-1.5,-1.365)	-1.431(-1.5,-1.362)	-1.436(-1.515,-1.357)
α_{23}^2	-1.369(-1.436,-1.307)	-1.367(-1.434,-1.304)	-1.363(-1.43,-1.301)	-1.361(-1.429,-1.294)
α_{24}^2	-1.424(-1.484,-1.366)	-1.422(-1.481,-1.363)	-1.42(-1.48,-1.359)	-1.423(-1.489,-1.357)
α_{25}^2	-1.432(-1.507,-1.361)	-1.43(-1.506,-1.359)	-1.423(-1.498,-1.355)	-1.413(-1.488,-1.344)
α_{26}^2	-1.511(-1.576,-1.446)	-1.51(-1.576,-1.44[illegible])	-1.507(-1.575,-1.441)	-1.498(-1.573,-1.424)
α_{27}^2	-1.583(-1.651,-1.513)	-1.584(-1.654,-1.513)	-1.581(-1.651,-1.512)	-1.58(-1.659,-1.502)
α_{28}^2	-1.564(-1.633,-1.495)	-1.566(-1.636,-1.497)	-1.563(-1.633,-1.493)	-1.56(-1.642,-1.481)
a_1	-3.344 (-3.547, -3.15)	-3.335 (-3.532, -3.142)	-3.298 (-3.485, -3.115)	-3.026 (-3.219, -2.824)
a_2	-0.352 (-0.378, -0.326)	-0.35 (-0.376, -0.324)	-0.34 (-0.365, -0.316)	-0.315 (-0.34, -0.289)

S4.2 Log-DNA intercept $\boldsymbol{\beta_{b,0}}$

The parameter $\beta_{b,0}$ is the intercept for the mean value of $l_{i,t}$ at $t = 1$, where we note that $\beta_{b,0}$ is on the log-DNA scale. Therefore close attention must be paid to what prior information on $\beta_{b,0}$ induces about the distribution on the baseline DNA scale. For example, a prior distribution of $\sim \mathrm{N}(0, 100^2)$ may be considered 'flat' on the log-scale, but is not so on the DNA scale. So instead consider the following prior distribution on $y = \exp(\beta_{b,0})$:

$$y \sim \mathrm{Unif}(0, b).$$

After a transformation of variables, we have the following induced distribution on $\beta_{b,0}$:

$$f(x) = \frac{1}{b}\exp(x),$$

for $x \in (-\infty, \log(b))$. This distribution on $\beta_{b,0}$ ($\sim$ Exp-Unif$(0, b)$) therefore induces a flat prior on DNA concentrations between 0 and some upper limit b.

In the rest of this section, we compare how this prior on $\beta_{b,0}$, for different values of b, affects posterior summaries. Tables S18, S19, and S20 show posterior means and 95% PCIs for the $l_{i,t}$ and covariate coefficients $\boldsymbol{\beta}_b$, $\boldsymbol{\beta}_w$ for the case studies in Sections 4.1, 4.2, and 4.3. We consider three different values for the upper DNA concentration limit in the prior, these being $b = \exp(20), \exp(30)$, and $\exp(100)$, and compare them to a data-informed normally distributed prior, where the prior mean is the average across all sites of the back-transformed CT values associated with $l_{i,1}$.

Generally we can see that the posterior distributions are not sensitive to the choice of upper limit b. We advise that this upper limit be set to several magnitudes larger than the highest expected DNA concentration (so as not to truncate posteriors at the upper limit). For example, in Table S18, the limit $b = \exp(20)$ is of magnitude roughly 200 times greater than the largest observed DNA concentration in the data.

Table S18: Posterior means and 95% PCIs for the $l_{i,t}$ and covariate coefficients $\boldsymbol{\beta}_b$ and $\boldsymbol{\beta}_w$ for the case study from Section 4.1 under different prior specification for $\beta_{b,0}$.

	$\sim \mathrm{N}(11.317, 1)$	$b = \exp(20)$	$b = \exp(30)$	$b = \exp(100)$
l_1	9.378(7.659,11.06)	9.067(7.331,10.738)	9.067(7.373,10.764)	9.065(7.362,10.761)
l_2	12.829(11.787,13.87)	12.498(11.444,13.548)	12.644(11.575,13.701)	12.633(11.574,13.692)
l_3	12.518(11.322,13.713)	12.356(11.175,13.555)	12.339(11.139,13.537)	12.335(11.151,13.524)
l_4	10.017(8.849,11.178)	9.73(8.534,10.915)	9.719(8.522,10.904)	9.71(8.526,10.89)
l_5	9.21(7.615,10.764)	8.88(7.296,10.447)	8.896(7.302,10.459)	8.886(7.308,10.422)
l_6	6.869(5.338,8.344)	6.727(5.187,8.183)	6.752(5.225,8.207)	6.748(5.235,8.216)
l_7	9.278(7.914,10.649)	9.025(7.666,10.419)	9.005(7.617,10.396)	9.001(7.645,10.378)
l_8	7.09(5.7,8.474)	6.887(5.482,8.277)	6.866(5.465,8.275)	6.851(5.455,8.233)
l_9	5.995(3.682,8.326)	5.895(3.57,8.228)	5.876(3.559,8.204)	5.892(3.64,8.188)
l_{10}	8.037(6.535,9.571)	7.893(6.392,9.416)	7.87(6.349,9.403)	7.858(6.359,9.359)
l_{11}	11.134(9.945,12.318)	11.008(9.845,12.174)	10.997(9.82,12.188)	10.994(9.795,12.172)
l_{12}	6.212(4.543,7.844)	5.876(4.18,7.511)	5.884(4.192,7.525)	5.871(4.205,7.486)
l_{13}	9.815(8.3,11.339)	9.673(8.195,11.157)	9.641(8.128,11.134)	9.637(8.119,11.147)
l_{14}	5.191(3.873,6.546)	4.89(3.543,6.265)	4.883(3.537,6.236)	4.859(3.508,6.216)
l_{15}	6.731(4.701,8.654)	6.364(4.336,8.279)	6.372(4.334,8.298)	6.358(4.375,8.252)
l_{16}	7.568(6.309,8.824)	7.321(6.059,8.589)	7.317(6.061,8.601)	7.298(6.036,8.597)
l_{17}	11.206(9.885,12.552)	10.979(9.649,12.31)	10.963(9.625,12.321)	10.96(9.631,12.289)
l_{18}	11.075(9.805,12.343)	10.885(9.601,12.187)	10.826(9.568,12.102)	10.847(9.573,12.134)
l_{19}	6.656(5.141,8.169)	6.492(4.991,7.986)	6.463(4.959,7.971)	6.443(4.96,7.979)
l_{20}	7.575(6.342,8.806)	7.27(6.004,8.531)	7.271(6.011,8.525)	7.259(5.997,8.51)
$\boldsymbol{\beta}_b[1]$	-0.985(-2.482,0.533)	-0.617(-2.123,0.907)	-0.617(-2.138,0.906)	-0.612(-2.102,0.931)
$\boldsymbol{\beta}_b[2]$	0.594(-0.922,2.095)	0.992(-0.573,2.533)	0.988(-0.565,2.511)	1.003(-0.578,2.521)
$\boldsymbol{\beta}_b[3]$	-0.584(-2.039,0.888)	-0.186(-1.72,1.306)	-0.175(-1.686,1.336)	-0.172(-1.68,1.333)
$\boldsymbol{\beta}_w[1]$	0.449(0.154,0.744)	0.436(0.144,0.728)	0.438(0.146,0.733)	0.434(0.141,0.728)
$\boldsymbol{\beta}_w[2]$	0.033(-0.553,0.613)	0.04(-0.546,0.629)	0.04(-0.549,0.635)	0.035(-0.547,0.609)
$\boldsymbol{\beta}_w[3]$	0.4(-0.408,1.263)	0.46(-0.331,1.312)	0.446(-0.35,1.302)	0.446(-0.348,1.285)
$\boldsymbol{\beta}_w[4]$	-0.026(-0.281,0.232)	-0.039(-0.292,0.217)	-0.04(-0.295,0.217)	-0.041(-0.294,0.217)
$\boldsymbol{\beta}_w[5]$	0.104(-0.68,0.884)	0.357(-0.448,1.16)	0.368(-0.452,1.172)	0.378(-0.431,1.184)
$\boldsymbol{\beta}_w[6]$	0.268(-0.618,1.155)	0.475(-0.443,1.378)	0.487(-0.428,1.394)	0.496(-0.421,1.398)
$\boldsymbol{\beta}_w[7]$	-0.396(-1.21,0.403)	-0.229(-1.054,0.6)	-0.216(-1.044,0.612)	-0.208(-1.035,0.611)

Table S19: Posterior means and 95% PCIs for the $l_{i,t}$ and covariate coefficients $\boldsymbol{\beta}_b$ and $\boldsymbol{\beta}_w$ for the case study from Section 4.2 under different prior specification for $\beta_{b,0}$.

	$\sim$ N(4.693, 1)	$b = \exp(20)$	$b = \exp(30)$	$b = \exp(100)$
$l_{1,1}$	4.962(4.138,5.785)	4.917(4.06,5.776)	4.917(4.076,5.759)	4.921(4.085,5.763)
$l_{1,2}$	7.094(6.13,8.048)	7.088(6.124,8.059)	7.111(6.166,8.075)	7.095(6.137,8.047)
$l_{1,3}$	5.549(4.385,6.713)	5.573(4.418,6.733)	5.581(4.442,6.723)	5.567(4.41,6.72)
$l_{1,4}$	5.322(4.15,6.521)	5.331(4.151,6.533)	5.345(4.178,6.508)	5.338(4.176,6.511)
$l_{1,5}$	4.433(3.383,5.483)	4.431(3.367,5.496)	4.433(3.402,5.485)	4.429(3.372,5.469)
$l_{1,6}$	6.318(5.322,7.313)	6.297(5.284,7.305)	6.318(5.336,7.293)	6.307(5.317,7.308)
$l_{1,7}$	8.391(7.423,9.343)	8.359(7.394,9.332)	8.376(7.431,9.33)	8.379(7.431,9.326)
$l_{1,8}$	10.839(9.866,11.812)	10.81(9.822,11.797)	10.825(9.846,11.804)	10.817(9.845,11.79)
$l_{1,9}$	10.471(9.303,11.656)	10.461(9.284,11.659)	10.46(9.315,11.642)	10.452(9.279,11.647)
$l_{1,10}$	10.037(9.15,10.931)	10.026(9.12,10.921)	10.057(9.172,10.943)	10.048(9.166,10.939)
$l_{1,11}$	5.502(4.585,6.423)	5.498(4.547,6.442)	5.505(4.586,6.428)	5.503(4.586,6.42)
$l_{1,12}$	6.155(5.215,7.093)	6.148(5.204,7.094)	6.136(5.175,7.074)	6.094(5.062,7.063)
$l_{2,1}$	2.809(1.908,3.694)	2.731(1.813,3.654)	2.74(1.816,3.631)	2.749(1.83,3.655)
$l_{2,2}$	-0.424(-2.925,1.599)	-0.466(-3.113,1.591)	-0.452(-2.936,1.524)	-0.479(-3.101,1.518)
$l_{2,3}$	0.313(-1.516,1.943)	0.272(-1.834,1.945)	0.289(-1.409,1.899)	0.33(-1.43,1.937)
$l_{2,4}$	0.007(-1.172,1.054)	0.007(-1.182,1.066)	0.005(-1.191,1.057)	0.023(-1.156,1.081)
$l_{2,5}$	2.234(1.126,3.301)	2.203(1.106,3.268)	2.242(1.145,3.3)	2.247(1.156,3.318)
$l_{2,6}$	2.931(1.506,4.335)	2.972(1.513,4.387)	2.961(1.543,4.368)	2.972(1.539,4.386)
$l_{2,7}$	4.363(3.344,5.378)	4.355(3.324,5.37)	4.36(3.334,5.374)	4.38(3.356,5.383)
$l_{2,8}$	10.054(8.94,11.159)	10.051(8.946,11.171)	10.036(8.951,11.131)	10.058(8.937,11.158)
$l_{2,9}$	8.827(7.859,9.797)	8.815(7.84,9.794)	8.832(7.878,9.79)	8.817(7.855,9.776)
$l_{2,10}$	5.292(4.375,6.219)	5.285(4.338,6.224)	5.29(4.36,6.192)	5.3(4.378,6.218)
$l_{2,11}$	5.018(4.052,5.985)	5.025(4.032,6.017)	5.003(4.04,5.969)	5.006(4.038,5.966)
$l_{2,12}$	4.821(3.6,6.041)	4.826(3.589,6.052)	4.82(3.602,6.025)	4.835(3.619,6.029)
$\boldsymbol{\beta}_w[1]$	0.402(-0.031,0.834)	0.416(-0.028,0.85)	0.414(-0.018,0.841)	0.411(-0.026,0.843)
$\boldsymbol{\beta}_w[2]$	-0.04(-0.304,0.228)	-0.034(-0.308,0.239)	-0.031(-0.294,0.234)	-0.032(-0.298,0.232)
$\boldsymbol{\beta}_w[3]$	0.15(-0.46,0.774)	0.144(-0.467,0.777)	0.157(-0.445,0.777)	0.149(-0.459,0.772)

Table S20: Posterior means and 95% PCIs for the $l_{i,t}$ and covariate coefficients $\boldsymbol{\beta}_b$ and $\boldsymbol{\beta}_w$ for the case study from Section 4.3 under different prior specification for $\beta_{b,0}$.

	$\sim \mathrm{N}(-8.122, 1)$	$b = \exp(20)$	$b = \exp(30)$	$b = \exp(100)$
$l_{1,1}$	-8.63(-9.802,-7.471)	-8.609(-9.852,-7.37)	-8.608(-9.859,-7.366)	-8.662(-9.926,-7.399)
$l_{1,2}$	-8.9(-10.084,-7.727)	-8.886(-10.089,-7.702)	-8.861(-10.079,-7.673)	-8.911(-10.143,-7.676)
$l_{1,3}$	-7.866(-9.051,-6.676)	-7.854(-9.047,-6.66)	-7.915(-9.116,-6.729)	-7.937(-9.168,-6.713)
$l_{1,4}$	-9.072(-10.245,-7.914)	-9.066(-10.26,-7.869)	-9.039(-10.246,-7.855)	-9.094(-10.348,-7.881)
$l_{1,5}$	-8.322(-9.301,-7.35)	-8.308(-9.324,-7.3)	-8.294(-9.313,-7.302)	-8.369(-9.401,-7.326)
$l_{1,6}$	-9.628(-10.477,-8.784)	-9.634(-10.503,-8.777)	-9.662(-10.515,-8.802)	-9.745(-10.643,-8.853)
$l_{1,7}$	-8.224(-9.203,-7.258)	-8.203(-9.189,-7.218)	-8.206(-9.199,-7.224)	-8.233(-9.261,-7.216)
$l_{1,8}$	-8.066(-8.899,-7.232)	-8.055(-8.918,-7.207)	-8.102(-8.964,-7.25)	-7.84(-8.74,-6.945)
$l_{1,9}$	-6.046(-7.215,-4.869)	-6.024(-7.204,-4.852)	-6.352(-7.556,-5.151)	-6.046(-7.25,-4.83)
$l_{1,10}$	-5.333(-6.513,-4.163)	-5.309(-6.498,-4.146)	-7.687(-8.923,-6.451)	-5.381(-6.59,-4.182)
$l_{1,11}$	-6.723(-7.872,-5.595)	-6.706(-7.864,-5.556)	-6.937(-8.111,-5.77)	-6.712(-7.914,-5.52)
$l_{1,12}$	-6.209(-7.361,-5.075)	-6.196(-7.359,-5.028)	-6.221(-7.378,-5.075)	-6.231(-7.424,-5.04)
$l_{1,13}$	-5.982(-7.136,-4.843)	-5.967(-7.126,-4.815)	-5.976(-7.13,-4.824)	-6.009(-7.202,-4.824)
$l_{1,14}$	-5.809(-6.79,-4.828)	-5.79(-6.805,-4.793)	-5.819(-6.845,-4.813)	-5.851(-6.893,-4.806)
$l_{1,15}$	-8.365(-9.553,-7.17)	-8.363(-9.572,-7.173)	-8.341(-9.554,-7.139)	-8.383(-9.63,-7.15)
$l_{1,16}$	-10.453(-11.496,-9.435)	-10.446(-11.515,-9.397)	-10.454(-11.523,-9.4)	-10.477(-11.565,-9.396)
$l_{1,17}$	-11.303(-12.402,-10.219)	-11.305(-12.43,-10.19)	-11.256(-12.393,-10.127)	-11.352(-12.496,-10.207)
$l_{1,18}$	-11.071(-12.443,-9.739)	-11.05(-12.427,-9.716)	-11.065(-12.408,-9.751)	-11.168(-12.575,-9.793)
$l_{1,19}$	-10.677(-12.071,-9.344)	-10.665(-12.057,-9.303)	-10.647(-12.052,-9.279)	-10.772(-12.206,-9.367)
$\boldsymbol{\beta}_b[1]$	0.455(-0.531,1.396)	0.499(-0.617,1.585)	0.739(-0.356,1.77)	0.52(-0.618,1.594)
$\boldsymbol{\beta}_b[2]$	0.025(-0.486,0.544)	0.02(-0.502,0.531)	0.034(-0.462,0.522)	0.025(-0.489,0.535)
$\boldsymbol{\beta}_w[1]$	0.035(-0.664,0.73)	0.024(-0.684,0.732)	0.022(-0.692,0.744)	0.027(-0.709,0.754)
$\boldsymbol{\beta}_w[2]$	0.345(-0.359,1.045)	0.332(-0.38,1.044)	0.334(-0.376,1.055)	0.341(-0.395,1.068)
$\boldsymbol{\beta}_w[3]$	-0.472(-1.089,0.142)	-0.476(-1.097,0.141)	-0.476(-1.096,0.135)	-0.502(-1.149,0.15)
$\boldsymbol{\beta}_w[4]$	-0.253(-0.863,0.36)	-0.259(-0.869,0.358)	-0.244(-0.858,0.366)	-0.272(-0.911,0.368)
$\boldsymbol{\beta}_w[5]$	-1.075(-1.677,-0.467)	-1.08(-1.693,-0.462)	-1.106(-1.728,-0.481)	-1.077(-1.716,-0.437)
$\boldsymbol{\beta}_w[6]$	-0.576(-1.178,0.039)	-0.574(-1.173,0.031)	-0.612(-1.219,0.002)	-0.557(-1.185,0.078)
$\boldsymbol{\beta}_w[7]$	0.397(-0.201,0.992)	0.399(-0.201,1.007)	0.383(-0.214,0.988)	0.417(-0.207,1.043)
$\boldsymbol{\beta}_w[8]$	-0.651(-1.276,-0.021)	-0.649(-1.281,-0.015)	-0.653(-1.283,-0.021)	-0.6(-1.249,0.057)
$\boldsymbol{\beta}_w[9]$	-0.538(-1.158,0.09)	-0.533(-1.151,0.091)	-0.54(-1.165,0.093)	-0.368(-1.013,0.284)

S5 Sample Contamination

In the model presented in Section 2, we assumed that contamination and inhibition occur on the level of the replicates within the lab. However, contamination and inhibition can also occur at the sample collection stage. This can occur, for example, due to contamination of collection equipment, or the collection of environmental material that interferes with the PCR process. In this section, we investigate the effect of ignoring this source of contamination and inhibition on model output through simulation. We simulate the data as in Section 3, with the exception of adding in the possibility of contamination or inhibition of collected samples. We denote by p_c^s and p_h^s the probabilities of contamination and inhibition of samples respectively. We assume that a sample cannot simultaneously experience contamination and inhibition. A contaminated sample has an additional DNA concentration, denoted λ^s, drawn from $\mathrm{N}(\lambda_0^s, (\sigma^s)^2)$. For inhibited samples, we reduce the amount of DNA in the sample by some proportion δ^s. In the following simulations we use the same model parameters as in Section 3, let $(p_c, p_h) = (0.05, 0.1)$, and consider the study design where $M = 5$, and $K = 5$. We then consider four possible scenarios, alternating between low and high probabilities of contamination and inhibition, and low and high amounts of contamination and inhibition. The simulation parameters are:

- S1: $(p_c^s, p_h^s) = (0.05, 0.1)$, $(\lambda_0^s, \sigma^s) = (3000, 1000)$, $\delta^s = 0.9$.
- S2: $(p_c^s, p_h^s) = (0.05, 0.1)$, $(\lambda_0^s, \sigma^s) = (30, 10)$, $\delta^s = 0.3$.
- S3: $(p_c^s, p_h^s) = (0.01, 0.02)$, $(\lambda_0^s, \sigma^s) = (3000, 1000)$, $\delta^s = 0.9$.
- S4: $(p_c^s, p_h^s) = (0.01, 0.02)$, $(\lambda_0^s, \sigma^s) = (30, 10)$, $\delta^s = 0.3$.

We compare results to those from Section 3.1 where $(p_c^s, p_h^s) = (0, 0)$, denoted simulation $S0$.

Table S21 shows the MSE, 95% PCIs and mean coverage for the $l_{i,t}$ for the four simulation scenarios compared to the non-sample contamination/inhibition baseline. Table S22 shows the MB, mean width of 95% PCIs and mean intervals containing zero for the covariate coefficients and probabilities of replicate contamination and inhibition p_c and p_h. Table S21 shows that as the probability and amount of contamination and inhibition of collected samples increases, then so too does the MSE of posterior means and the posterior 95% PCIs for $l_{i,t}$. Table S22 shows that the mean bias and width of 95% PCIs also increases, but that for small probabilities and amounts of sample contamination and inhibition that these are comparable to results under the baseline S0. If field negatives are collected, then these can be incorporated into the model. Field negatives could indicate the rate and magnitude of contamination effects during the collection stages of a particular sampling occasion.

Table S21: Mean square error (MSE), mean range of 95% PCIs (R), and mean coverage of $l_{i,t}$ (C) for five simulation scenarios S0 to S4. Probability of contamination and inhibition $(p_c, p_h) = (0.05, 0.1)$, $M = 5$, and $K = 5$.

	S0	S1	S2	S3	S4
MSE	0.249	0.467	0.455	0.301	0.317
R	1.790	2.225	1.795	1.899	1.811
C	0.950	0.928	0.928	0.943	0.941

Table S22: Mean bias (MB), mean width of the 95% PCIs (R), and the mean number of intervals containing zero (Z) of the covariate coefficients $\boldsymbol{\beta}_b, \boldsymbol{\beta}_w$, and probabilities of contamination and inhibition p_c and p_h respectively under five simulation scenarios S0 to S4. Probability of contamination and inhibition $(p_c, p_h) = (0.05, 0.1)$, $M = 5$, and $K = 5$.

	S0	S1	S2	S3	S4
$\boldsymbol{\beta}_b[1]$					
MB	0.005	-0.029	-0.015	-0.001	0.001
R	0.277	0.315	0.276	0.282	0.274
Z	0.000	0.000	0.000	0.000	0.000
$\boldsymbol{\beta}_b[2]$					
MB	0.011	0.060	0.074	0.058	0.032
R	0.535	0.618	0.536	0.556	0.538
Z	0.000	0.000	0.000	0.000	0.000
$\boldsymbol{\beta}_w[1]$					
MB	0.008	-0.006	-0.029	0.011	0.001
R	0.152	0.208	0.156	0.164	0.154
Z	0.000	0.000	0.000	0.000	0.000
$\boldsymbol{\beta}_w[2]$					
MB	-0.011	0.031	0.027	0.000	0.002
R	0.298	0.407	0.304	0.321	0.300
Z	0.000	0.000	0.000	0.000	0.000
p_c					
MB	-0.021	-0.017	-0.021	-0.020	-0.021
R	0.007	0.009	0.007	0.007	0.007
Z	-	-	-	-	-
p_h					
MB	-0.003	-0.007	-0.005	-0.002	-0.004
R	0.016	0.015	0.015	0.014	0.014
Z	-	-		-	-

References

[1] Biggs, J., Ewald, N., Valentini, A., Gaboriaud, C., Griffiths, R., Foster, J., Wilkinson, J., Arnett, A., Williams, P., Dunn, F.: Analytical and methodological development for improved surveillance of the great crested newt. appendix 5. technical advice note for field and laboratory sampling of great crested newt (triturus cristatus) environmental dna. Technical report, Freshwater Habitats Trust, Oxford (2014)

[2] Buxton, A.S., Groombridge, J.J., Zakaria, N.B., Griffiths, R.A.: Seasonal variation in environmental dna in relation to population size and environmental factors. Scientific Reports **7**(1), 46294 (2017) https://doi.org/10.1038/srep46294

[3] Bates, D., Mächler, M., Bolker, B., Walker, S.: Fitting linear mixed-effects models using lme4. Journal of Statistical Software **67**(1), 1–48 (2015) https://doi.org/10.18637/jss.v067.i01

[4] Espe, M.B., Johnston, M., Blankenship, S.M., Dean, C.A., Bowen, M.D., Schultz, A., Schumer, G.: The artemis package for environmental dna analysis in r. Environmental DNA **4**(3), 523–532 (2022) https://doi.org/10.1002/edn3.277

[5] Knowles, J.E., Frederick, C.: merTools: Tools for Analyzing Mixed Effect Regression Models. (2024). R package version 0.6.2. https://CRAN.R-project.org/package=merTools

[6] Plummer, M., Best, N., Cowles, K., Vines, K.: Coda: Convergence diagnosis and output analysis for mcmc. R News **6**(1), 7–11 (2006)

[7] R Core Team: R: A Language and Environment for Statistical Computing. R Foundation for Statistical Computing, Vienna, Austria (2018). R Foundation for Statistical Computing. https://www.R-project.org/

[8] Smyth, G., Dunn, P.K., Corty, R.W.: Dglm: Double Generalized Linear Models. (2023). R package version 1.8.6. https://CRAN.R-project.org/package=dglm

[9] Shelton, A.O., Ramón-Laca, A., Wells, A., Clemons, J., Chu, D., Feist, B.E., Kelly, R.P., Parker-Stetter, S.L., Thomas, R., Nichols, K.M., Park, L.: Environmental dna provides quantitative estimates of pacific hake abundance and distribution in the open ocean. Proceedings of the Royal Society B **289**(1971), 20212613 (2022) https://doi.org/10.1098/rspb.2021.2613
